# Astilbin Activates the Reactive Oxidative Species/PPARγ Pathway to Suppress Effector CD4^+^ T Cell Activities *via* Direct Binding With Cytochrome P450 1B1

**DOI:** 10.3389/fphar.2022.848957

**Published:** 2022-05-16

**Authors:** Shizhen Ding, Guotao Lu, Biying Wang, Jie Xiang, Chunxia Hu, Zhijie Lin, Yanbing Ding, Weiming Xiao, Weijuan Gong

**Affiliations:** ^1^ Department of Gastroenterology, Affiliated Hospital of Yangzhou University, Yangzhou University, Yangzhou, China; ^2^ Department of Immunology, School of Medicine, Yangzhou University, Yangzhou, China; ^3^ Department of Pharmacology, School of Medicine, Yangzhou University, Yangzhou, China; ^4^ Jiangsu Key Laboratory of Integrated Traditional Chinese and Western Medicine for Prevention and Treatment of Senile Diseases, Yangzhou University, Yangzhou, China; ^5^ Jiangsu Key Laboratory of Zoonosis, Jiangsu Co-innovation Center for Prevention and Control of Important Animal Infectious Diseases and Zoonoses, Yangzhou University, Yangzhou, China

**Keywords:** astilbin, ROS, CD4^+^T cell, cytochrome P450 1B1, anti-inflammation, PPARγ

## Abstract

Astilbin, as a compound of flavonoids, exerts anti-inflammation, antioxidation, and immune-suppression activities. Decreased activation of NF-κB and p38 MAPK and increased activation of SOCS3 and AMPK have been found in astilbin-treated cells. However, what molecules are docked by astilbin to initiate signaling cascades and result in functional changes remains unknown. In the study, we found that astilbin efficiently suppressed TNF-α production and increased CCR9 and CD36 expression of CD4^+^ T cells. *In vivo* administration of astilbin repressed the occurrence of type 1 diabetes mellitus in non-obese diabetic mice. The PPARγ/SOCS3, PPARγ/PTEN, and PPARγ/AMPK signaling pathways were substantially activated and played key roles in astilbin-induced downregulation of CD4^+^ T cell functions. Transcriptome sequencing results confirmed the changes of signaling molecules involved in the immune system, inflammatory responses, and indicated variations of multiple enzymes with oxidant or antioxidant activities. Astilbin directly induced cytoplasmic ROS production of CD4^+^ T cells *ex vivo*, but had no effects on mitochondrial ROS and mitochondrial weight. When cellular ROS was depleted, astilbin-treated CD4^+^ T cells remarkably reversed the expression of TNF-α, IFN-γ, CCR9, CD36, and signaling molecules (PPARγ, PTEN, p-AMPK, and SOCS3). Based on bioinformatics, two P450 enzymes (CYP1B1 and CYP19A1) were selected as candidate receptors for astilbin. CYP1B1 was identified as a real docking protein of astilbin in ROS production by AutoDock Vina software analysis and surface plasmon resonance assay. Collectively, astilbin downregulates effector CD4^+^ T cell activities *via* the CYP1B1/ROS/PPARγ pathway, which firmly supports its potential use in the treatment of inflammation.

## Introduction

Astilbin is a flavonoid compound that can be extracted from *Smilax glabra* rhizomes, *Hypericum perforatum*, and various French vines (0.78–15.12 mg/dl) (Sharma et al., 2020). It possesses strong anti-inflammation, immunosuppression, anti-microorganism, and antioxidation activities (Panche et al., 2016) and plays a therapeutic role in various disorders, such as diabetes (Perez-Najera et al., 2018), liver injury (Wang et al., 2004), lung injury (H. B. Zhang H B et al., 2017), cardiovascular infarction (Diao et al., 2014), depression (Lv et al., 2014), myasthenia gravis (Meng et al., 2016), neurodegenerative diseases (Wang et al., 2017; Zhu et al., 2019), renal dysfunction (Chen et al., 2018), contact hypersensitivity (Fei et al., 2005), psoriasis (Di et al., 2016), burn wound healing (Kimura et al., 2007), androgenic alopecia (Nagasawa et al., 2016), systemic lupus erythematosus (Guo et al., 2015), transplant arteriosclerosis (Zhao et al., 2009), and arthritis (Cai et al., 2003). Astilbin also inhibits the activities of angiotensin-converting enzyme, glucose-6-phosphatase, aldose reductase, α-glucosidase, horseradish peroxidase, and myeloperoxidase and amplifies lipoprotein lipase activity, thus exerting modulatory effects on blood pressure and metabolism (Sharma et al., 2020).

The mechanisms of astilbin-mediated anti-inflammation and immunosuppression are involved with downregulating the activities of macrophages, dendritic cells (Ding et al., 2014), and effector T cells and inducing regulatory B cells (Xu et al., 2020) and T cells (Han et al., 2020). Astilbin suppresses the function of inflammatory cells by inhibiting the PI3K/AKT, TLR4/MyD88/NF-κB (Li et al., 2019), and MAPK signaling pathways and promoting the expression of SOCS3 and activating AMPK, leading to less production of IL-1β, IL-6, TNF-α, IFN-γ, and MMPs (Sharma et al., 2020; Yang et al., 2021). Astilbin is also verified to have strong capacity for scavenging reactive oxidative species (ROS) directly or indirectly (Wang et al., 2019). Astilbin promotes Nrf2 nucleus translocation in skin cells (HaCaT) to reduce ROS accumulation, VEGF expression, and HaCaT cell proliferation (C. Zhang et al., 2017). However, what molecules have dock sites for astilbin and how astilbin modifies the actions of its receptors to induce downstream signaling events in cells, remains unknown.

Astilbin was firstly identified to inhibit effector CD4^+^ T cell function in concanavalin A-induced liver injury (Wang et al., 2004). Astilbin efficiently suppresses TNF-α and MMP-9 production and adhesion of CD4^+^ T cells, as well as Th17 differentiation (Meng et al., 2016). Given the key role of effector CD4^+^ T cells in inflammation-associated diseases, the accurate elucidation of the effects and mechanisms of astilbin on CD4^+^ T cells would promote its clinical application. In the present study, we profoundly investigated the molecular mechanisms behind the inhibitory effects of astilbin on effector CD4^+^ T cells and found that CYP1B1 was one dock molecule of astilbin associated with its ability to modulate CD4^+^ T cell function.

## Materials and Methods

### Animals and Reagents

Non-obese diabetic (NOD) mice and *Nrf2* ^*−/−*^ mice were obtained from the Model Animal Research Center of Nanjing University (Nanjing, China). C57BL/6 mice were from Comparative Medical Center of Yangzhou University (Yangzhou, China). Experiments used 6–12-week-old and sex-matched mice, unless otherwise indicated. All animal care and handling procedures were conducted in accordance with the protocols approved by the Institutional Animal Care and Use Committee (IACUC) at Yangzhou University (Yangzhou, China).

The following reagents were used: Astilbin (HY-N0509); 1-aminobenzotriazole (ABT, HY-103389), a nonspecific and irreversible inhibitor of CYP enzymes; etomoxir (HY-50202), an inhibitor of CPT1α; SF1670 (HY-15842), an inhibitor of PTEN; N-Acetyl-L-cysteine (NAC, HY-B0215), a GSH-related antioxidant; GW9662 (HY-16578), a potent and selective PPARγ antagonist; and dorsomorphin (Compound C, HY-13418A), a selective and ATP-competitive AMPK inhibitor (MedChemExpress, NJ, United States).

### Cell Culture

CD3^+^ CD4^+^ cells were isolated from the spleen by magnetic bead sorting (Miltenyi Biotec GmbH, BG, Germany). Isolated CD4^+^ T cells and unlabeled cells were counted and then cultured in RPMI containing 10% FBS and penicillin/streptomycin in a humidified atmosphere containing 5% CO_2_ at 37°C. Cells were further stimulated *in vitro* with astilbin, with or without IL-12 (20 U/ml), plate-bound anti-CD3 (2 μg/ml) and soluble anti-CD28 (1 μg/ml) antibodies, GW9662, NAC, SPF1670, dorsomorphin, ABT, and etomoxir according to the experimental design. After 12 h, 24 h, or 2 days, cells were harvested for flow cytometric analysis.

### Flow Cytometry

Cells were stained with the indicated antibodies for 30 min at 4°C. The following antibodies for flow cytometry were obtained from BioLegend, CA, United States: anti-mouse CD4 (GK1.5), anti-mouse CCR6 (29-2L17), anti-mouse CCR9 (9B1), anti-mouse CD36 (HM36), and anti-human CD4 (OKT4). For intracellular cytokine analysis, cells were stimulated with PMA and ionomycin for 30 min and treated with brefeldin A for 4 h before staining Fixation/Permeabilization Solution Kit (BD, NJ, United States) followed by anti-mouse TNF-α (MP6-XT22, BioLegend, CA, United States), anti-mouse IFN-γ (XMG1.2, BD, NJ, United States), anti-human TNF-α (Mab11, BioLegend, CA, United States), or anti-human IFN-γ (4S.B3, BioLegend, CA, United States). Cell apoptosis was detected with an Annexin V staining kit (BD, NJ, United States) according to the manufacturer’s instructions. For cell proliferation, cells were labeled with 5 μM CFDA-SE (CFSE) (Invitrogen, CA, United States). Seventy-two hours later, they were collected and analyzed by flow cytometry. Acquisition was carried out on FACSVerse (BD, NJ, United States), and analysis was performed with FlowJo v10 software.

### Evaluation of Type 1 Diabetes Mellitus

NOD mice were randomly divided into three groups, and each group was intraperitoneal injected twice weekly with PBS or astilbin (50, 100 mg/kg) for 7 weeks. Body weight and blood glucose of NOD mice were monitored once a week until the end of the observation period. NOD mice were characterized as diabetic when blood glucose readings were >13.9 mmol/L for two consecutive measurements. The indexes of dead mice were shown as the last blood glucose or weight data. At the end of the observation period, mice were euthanized by cervical dislocation, and pancreatic CD4^+^ T cells were measured by flow cytometry.

### Western Blot

Total protein was extracted and boiled for 5 min, resolved by electrophoresis, and transferred to PVDF membranes. Blots were blocked for 1 h with 5% milk in TBST followed by incubation with antibodies (dilution 1:500 to 1:2000) at 4°C overnight. The following antibodies were used for western blot: ACC (Cat# 3676, RRID:AB_2219397), p-ACC (Cat# 11818, RRID:AB_2687505), HKII (Cat# 2867, RRID:AB_2232946), mTOR (Cat# 2983, RRID:AB_2105622), p-mTOR (Cat# 5536, RRID:AB_10691552), Akt (Cat# 4691, RRID:AB_915783), p-Akt (Cat# 4060, RRID:AB_2315049), AMPK (Cat# 5831, RRID:AB_10622186), p-AMPK (Cat# 50081, RRID:AB_2799368), LKB1 (Cat# 3047, RRID:AB_2198327), p-LKB1 (Cat# 3482, RRID:AB_2198321), PTEN (Cat# 9188, RRID:AB_2253290), PPAR-γ (Cat# 2443, RRID:AB_823598), PI3K p110α (Cat# 4249, RRID:AB_2165248), β-actin (Cat# 4970, RRID:AB_2223172), NF-κB p65 (Cat# 8242, RRID:AB_10859369), NF-κB p-p65 (Cat# 3033, RRID:AB_331284), p38 (Cat# 8690, RRID:AB_10999090), p-p38 (Cat# 4511, RRID:AB_2139682), JNK (Cat# 9252, RRID:AB_2250373), p-JNK (Cat# 4668, RRID:AB_823588), Erk (Cat# 4695, RRID:AB_390779), p-Erk (Cat# 4370, RRID:AB_2315112), Stat3 (Cat# 4904, RRID:AB_331269), p-Stat3 (Cat# 9145, RRID:AB_2491009), SOCS3 (Cat# 52113, RRID:AB_2799408) (Cell Signaling Technology, MA, United States), Glut1 (Cat# ab115730, RRID:AB_10903230), CPT1α (Cat# ab234111, RRID:AB_2864319), LPL (Cat# ab21356, RRID:AB_446221) (Abcam, Cambridge, Britain), CYP1B1 (Cat# 18505-1-A, RRID:AB_2878548) (Proteintech, IL, United States), CYP19A1 (Cat# YT1190, RRID:AB_2864736) (Immunoway, DE, United States) and P-PPARγ (abs130911, absin, Shanghai China). HRP-conjugated anti-rabbit (Cat# 7074, RRID:AB_2099233, Cell Signaling Technology, MA, United States) secondary antibodies were used at 1:5000 dilution for 1 h at room temperature. The bands were detected by developing with chemiluminescent HRP substrate (Thermo Scientific, MA, United States), and the intensity of bands was determined by imaging with a molecular imager (Bio-Rad, CA, United States). Bands quantified using the ImageJ software. All results were normalized to those of β-actin, which was used as a loading control, and phosphorylated proteins is compared to the total amount of proteins and housekeeping genes (β-actin).

### Detection of Reactive Oxidative Species and Mitochondria

To determine the mitochondrial mass and the levels of intracellular and mitochondrial ROS (mROS), the cells were incubated with 100 nM MitoTracker Green (Beyotime, Shanghai, China) (De Nicola et al., 2011), 10 μM DCFH-DA (Beyotime, Shanghai, China), and 10 μM MitoSOX Indicator (Thermo Fisher Scientific, MA, United States) for 15 min in a CO_2_ incubator at 37°C. After washing twice, the data were acquired on a FACSVerse (BD, NJ, United States) and analyzed with FlowJo v10 software.

### Transcriptome Sequencing

Total RNA was isolated from mouse CD4^+^ T cells with TRIzol (1 ml) reagent and purified using Quick-RNA Miniprep columns and RNase-free DNase digestion. RNA integrity was assessed with the Agilent 2100 Bioanalyzer (Agilent Technologies, CA, United States). RNA purity and quantity were evaluated with a NanoDrop 2000 spectrophotometer (Thermo Scientific, MA, United States). Only samples with RIN scores above nine and a minimum total RNA of 500 ng were used for library preparation. Then, the libraries were constructed using TruSeq Stranded mRNA LT Sample Prep Kit (Illumina, CA, United States) according to the manufacturer’s instructions. Subsequently, the transcriptome sequencing data were used for a series of analyses, including differential expression analysis, gene enrichment analysis, and expression level analysis.

### Prediction of Potential Targets of Astilbin

The potential targets of astilbin were predicted using the PharmMapper database (http://www.lilab-ecust.cn/pharmmapper/index.php) by performing Druggable Pharmacophore Models and SwissTargetPrediction (http://www.swisstargetprediction.ch/) on the basis of the principle that chemicals with similar structures may have similar functions. All the screened targets were input into the UniProt database (http://www.uniprot.org/) to exclude the same targets and non-Homo sapiens targets. The corresponding predicted target information obtained was collected and sorted according to fit score and probability.

### Docking Study

The chemical structure of astilbin was obtained from the PubChem compound database (http://pubchem.ncbi.nlm.nih.gov/) with ID 29838-67-3. The crystal structures of CYP1B1 and CYP19A1 proteins with PDB IDs 3PM0 and 3S79, respectively, were obtained from the Research Collaboratory for Structural Bioinformatics (RCSB) Protein Data Bank. The crystal water molecules, ligand atoms, and ions that were bound to the proteins were removed. Hydrogen atoms were subsequently added using AutoDock Tool program version 1.5.6. Molecular docking between astilbin and human cytochrome P450 1B1 (CYP1B1) was performed using AutoDock Vina on the basis of the Lamarckian genetic algorithm, which combines energy evaluation through grids of affinity potential to find a suitable binding position for a ligand on a given protein. The grid box for docking was positioned properly at the active binding site in the center. The genetic algorithm and its run were set to 1000, as the docking algorithms were set on default. Finally, the results were retrieved as binding energy, and docking with binding energies lower than −5 kcal/mol was selected as a significant binding event and was visualized using PyMOL version 1.5.0.3. software for obtaining hydrogen bond, hydrophobic, and electrostatic interactions.

### Surface Plasmon Resonance

SPR experiments were performed with a Biacore T200 (GE Healthcare, MA, United States). CYP1B1 coupling protein (ImmunoClone, NY, United States) was diluted in 10 mM sodium acetate (pH 5.0) and immobilized on the sensor chip CM7 by primary amine coupling kit (Biacore, GE Healthcare, MA, United States) to a final response level of approximately 6000 resonance units. A blank channel was used as the negative control. All data were collected at 25°C with HBS-EP running buffer (10 mM HEPES, 150 mM NaCl, 3 mM EDTA, 0.05% surfactant polysorbate 20, pH 7.4) at a flow rate of 30 μl/min. Concentration series of the original astilbin (two-fold dilutions starting from a top concentration of 200 μM) was injected over immobilized proteins for a contact time of 180 s and dissociation time of 300 s. The resulting data were fit to a 1:1 binding model using Biacore Evaluation Software v3.2, and the Kd value was determined.

### Knockdown of Cytochrome P450 1B1

Silencing of CYP1B1 in CD4^+^ T cells was achieved using lentivirus. The specific lentivirus CYP1B1-shRNA (5′-GCC​TGA​CCA​TTA​AGC​CCA​AGT​CTC​GAG​ACT​TGG​GCT​TAA​TGG​TCA​G GCTTTTT-3′) and negative control lentivirus shRNA were designed and synthesized by Genecreate, Wuhan, China. Lentiviral plasmid vectors and packaging vectors were co-transfected in 293T cells with Lipofectamine 3000 Reagent (Thermo Fisher, MA, United States). For lentiviral infection, CD4^+^ T cells were mixed with the virus and 10 μg/ml of polybrene (Sigma-Aldrich, MS, United States). The knockdown effect of CYP1B1 in CD4^+^ T cells was confirmed by RT-PCR and Western blot analysis.

### Statistical Analysis

All data are presented as the means ± SD or means ± SEM. Comparisons between two groups were assessed using the unpaired two-tailed student’s *t* test. Comparison of multiple groups was performed using one-way ANOVA or two-way ANOVA. All statistical tests were two-sided. Statistical differences are significant for *, *p* ≤ 0.05; **, *p* ≤ 0.01; ***, *p* ≤ 0.001.

## Results

### Astilbin Suppressed CD4^+^ T Cell Function Involved in Ameliorating Type 1 Diabetes Mellitus

**FIGURE 1:**
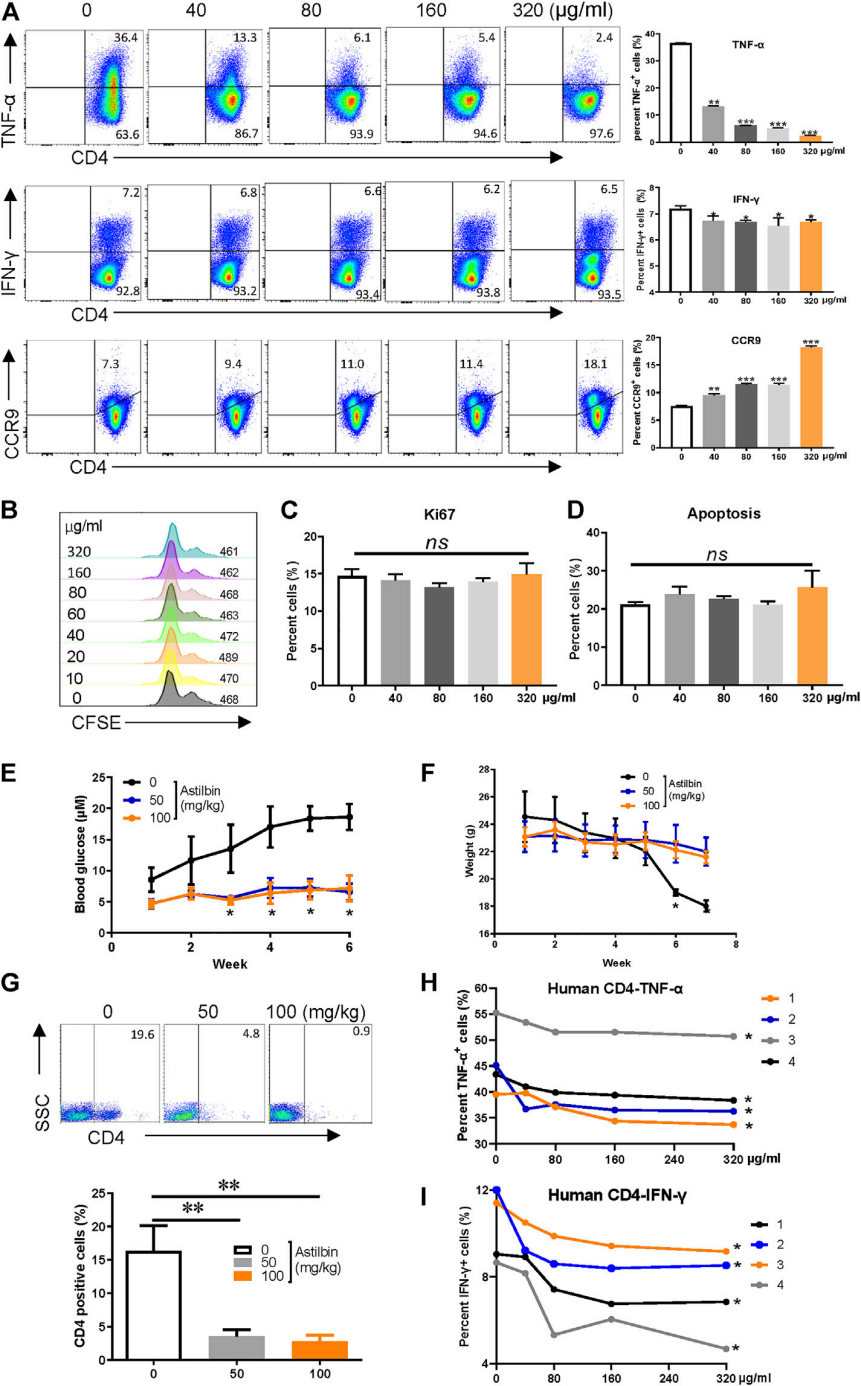
Astilbin decreases CD4^+^ T cell functions. **(A)** TNF-α^+^, IFN-γ^+^, and CCR9^+^ of CD4^+^T cells treated with various doses of astilbin. Mean ± SD; *n* = 3. **(B)** CD4^+^ T cell division by CFSE dilution. **(C)** Proliferative capacity of CD4^+^ T cells evaluated by Ki67. Mean ± SD; *n* = 3. **(D)** Apoptosis of astilbin-treated CD4^+^ T cells stained by Annexin V and PI. Mean ± SD; *n* = 3. **(E)** Blood glucose levels of NOD mice intraperitoneally injected with astilbin. Mean ± SEM; *n* = 10. **(F)** Weight curves of NOD mice. Mean ± SEM; *n* = 10. **(G)** Infiltrated CD4^+^ cells in the pancreas of NOD mice. Mean ± SD; *n* = 10. Variations of TNF-α **(H)** and IFN-γ **(I)** in human CD4^+^ T cells as treated by astilbin for 24 h. Mean ± SEM; *n* = 4. All *ex vivo* experiments were repeated at least three times. Experiments of astilbin-treated NOD mice were conducted twice. *p* values (*p ≤ 0.05; **p ≤ 0.01; ***p ≤ 0.001; ns, no significant difference) determined by one-way ANOVA **(A–C,G)**, two-way ANOVA **(E,F,H,I)**.

After the IC_50_ of astilbin on CD4^+^ T cells (181 μg/ml) was verified *ex vivo* (Supplementary Figure S1A), the effects of astilbin treatment at various doses on TNF-α and IFN-γ, two key inflammatory cytokines produced by effector CD4^+^ T cells, were checked. As shown in Figure 1A, TNF-α production could be substantially inhibited in a dose-dependent manner. Although IFN-γ secretion was also downregulated statistically, the extent was weak. CCR9 expression of lymphocytes indicates the gut-homing activity. Consistent with the effect of astilbin on NK1.1^-^ CD4^+^ NKG2D^+^ regulatory T cells (Han et al., 2020), the expression of CCR9 on CD4^+^ T cells was also enhanced with astilbin treatment in a dose-dependent manner. Unexpectedly, no changes in the expression of IL-10 and CCR6 in astilbin-treated CD4^+^ T cells were observed (Supplementary Figures S1B,C). Astilbin slightly decreased TGF-β1 secretion of CD4^+^ T cells (Supplementary Figure S1D). We also did not observe any effects of astilbin on the proliferation (Figures 1B,C) and apoptosis of CD4^+^ T cells at doses of 40–160 μg/ml (Figure 1D; Supplementary Figure S1E).

IFN-γ, IL-1β, and TNF-α production by autoreactive Th1 cells is involved in the destruction of insulin-secreting β-cells in type 1 diabetes (Kaufmann et al., 2019). Since we noted that astilbin could reduce IFN-γ and TNF-α in CD4^+^T cells, we then analyzed whether astilbin could repress the onset of type 1 diabetes in NOD mice by regulating CD4^+^T cells. Compared with control group, blood glucose levels and body weights were significantly controlled after the administration of astilbin (50 or 100 mg/kg) (Figures 1E,F). When mononuclear cells of the pancreas from mice were isolated, less CD4^+^ T cells infiltrated the pancreas of astilbin-administered mice (Figure 1G). These results demonstrated that astilbin ameliorated T1DM involved with the downregulation of CD4^+^ T cell activities. Finally, astilbin also efficiently decreased TNF-α and IFN-γ production of peripheral blood CD4^+^ T cells from human healthy individuals as displayed in Figures 1H,I. Therefore, we identified that astilbin exerted immune-suppressive activities *via* downregulating inflammatory cytokine secretions of CD4^+^ T cells.

### PPARγ/SOCS3 Pathway Involved in Astilbin-Mediated Inhibition

**FIGURE 2:**
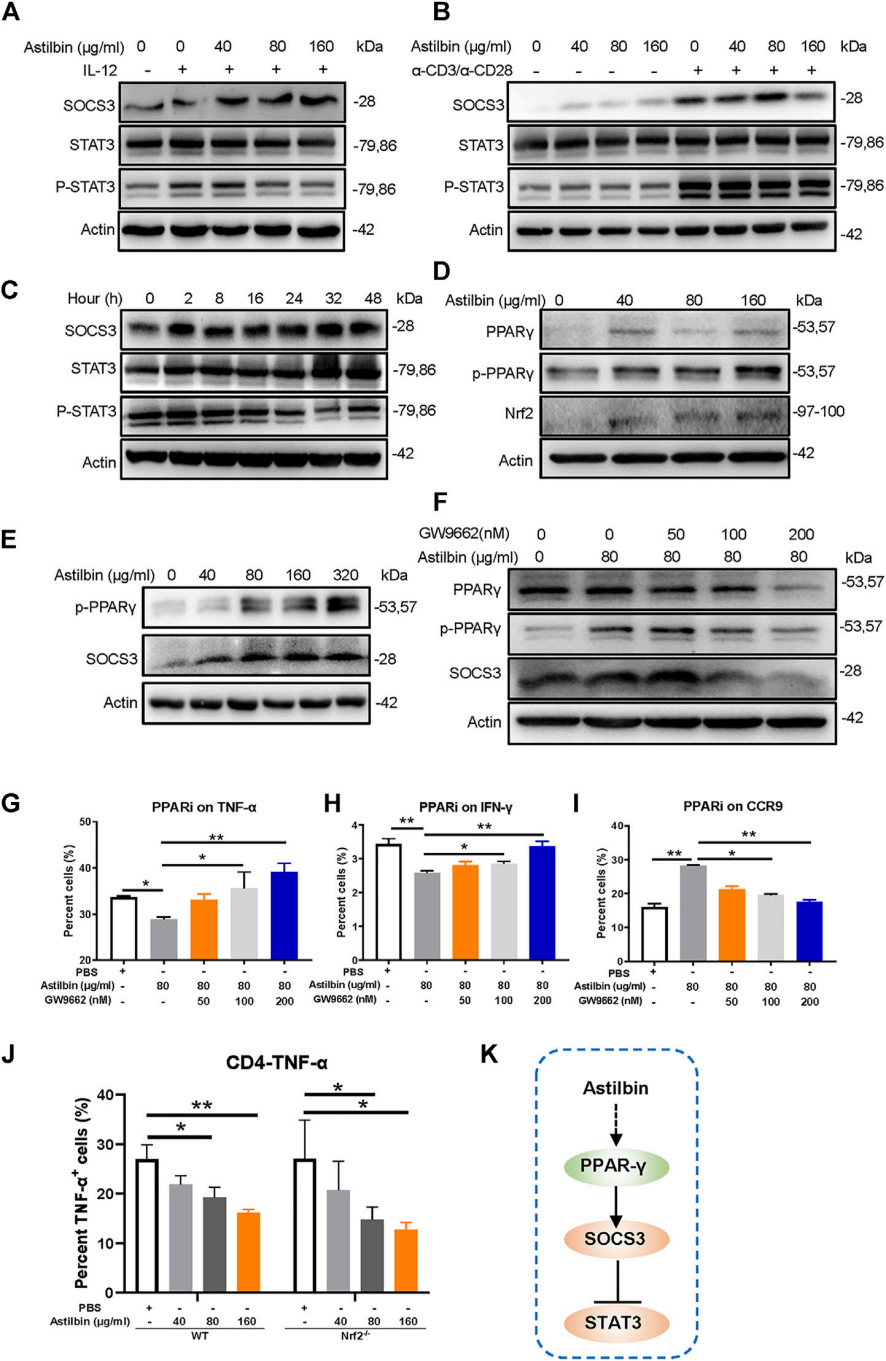
PPARγ/SOCS3 pathway in astilbin-treated CD4^+^ T cells. Effects of astilbin on the expression of SOCS3 and STAT3/p-STAT3 of unstimulated, IL-12- **(A)**, or α-CD3/α-CD28− **(B)** stimulated CD4^+^ T cells by Western blot analysis. **(C)** Effects of 80 μg/ml astilbin on SOCS3 and STAT3/p-STAT3 of activated CD4^+^ T cells at different time points. **(D)** Variations of PPARγ/p-PPARγ and Nrf2 in astilbin-treated CD4^+^ T cells. **(E)** Parallel expression of p-PPARγ and SOCS3 in astilbin-treated CD4^+^ T cells. **(F)** Inhibiting PPARγ by GW9662 decreased SOCS3 expression. Effects of GW9662 on TNF-α **(G)**, IFN-γ **(H)**, and CCR9 **(I)** expression in astilbin-treated CD4^+^ T cells. Mean ± SD; *n* = 3. **(J)** TNF-α production of astilbin-treated *Nrf2* ^*−/−*^ CD4^+^ T cells. Mean ± SD; *n* = 3. **(K)** Diagram of PPAR/SOCS3 pathway on CD4^+^ T cells by astilbin. Each experiment was repeated at least three times. The inhibitor of PPAR, GW9662, is abbreviated as PPARi. *p* values (*p ≤ 0.05; **p ≤ 0.01) determined by one-way ANOVA **(G–J)**.

Several studies have found that astilbin treatment increases the expression of SOCS3 (Wang et al., 2016; Han et al., 2020; Sharma et al., 2020). Variations of SOCS3 were first measured in freshly isolated, IL-12-activated, or α-CD3/α-CD28-activated CD4^+^ T cells treated by astilbin. Astilbin could increase SOCS3 expression of freshly isolated and activated CD4^+^ T cells. The critical role of SOCS3 is manifested by its binding to both the JAK kinase and the cytokine receptor, which can result in the inhibition of STAT3 phosphorylation. Consequently, as expected phosphorylated STAT3 (p-Y705) levels were downregulated in astilbin-treated CD4^+^ T cells (Figures 2A,B; Supplementary Figures S2A,B). However, when variations of SOCS3 were further detected after CD4^+^ T cells were treated by astilbin (80 μg/ml) at different time courses. SOCS3 expression was increased by 2-h coculture of astilbin and maintained high levels even at 48 h. In parallel, p-STAT3 level was decreased by 2-h treatment, maintained low levels at 8–16 h, and reversed to normal levels at 24 h (Figure 2C; Supplementary Figure S2C). This trend of p-STAT3 changes may indicate that STAT3 activation could be regulated by other factors, besides SOCS3.

In general, SOCS3 and STAT3 are mutually regulated in cells (Carow and Rottenberg, 2014). Considering the decreased activation of STAT3 and the upregulation of SOCS3, p-STAT3 might not have promoted SOCS3 transcription in astilbin-treated CD4^+^ T cells. SOCS3 transcription is also activated by PPARγ to prevent IL-17-derived cancer growth (Berger et al., 2013). We determined whether the increased SOCS3 of CD4^+^ T cells was promoted by PPARγ. As expected, total and phosphorylated PPARγ (p-PPARγ) were remarkably increased in astilbin-treated CD4^+^ T cells (Figure 2D; Supplementary Figure S2D). Moreover, astilbin treatment at different doses resulted in parallel variations of p-PPARγ and SOCS3 expression (Figure 2E; Supplementary Figure S2E) and the expression of SOCS3 decreased significantly after the addition of PPARγ inhibitor GW9662 in CD4^+^ T cells, which was similar to the changes of p-PPARγ (Figure 2F; Supplementary Figure S2F). Next, GW9662 was used simultaneously to analyze the effects on functional changes of CD4^+^ T cells, which were cotreated by astilbin. Treatment of GW9662 reversed TNF-α (Figure 2G) and IFN-γ (Figure 2H) production and decreased CCR9 expression (Figure 2I) of astilbin-treated CD4^+^ T cells (Supplementary Figure S2G).

Another prominent regulator of antioxidant signaling pathways, that is, positively associated with PPARγ regulation is nuclear factor erythroid 2-related factor 2 (Nrf2) (Annie-Mathew et al., 2021). We then tested the expression of Nrf2 in astilbin-treated CD4^+^T cells. Interestingly, Nrf2 was also enhanced by astilbin treatment (Figure 2D). However, when CD4^+^ T cells derived from *Nrf2* ^*−/−*^ mice were treated by astilbin, the decreased TNF-α production was not almost affected (Figure 2J; Supplementary Figure S2H), indicating that astilbin-mediated immunoregulatory effect was independent on Nrf2. Collectively, the above results demonstrated the key role of the PPARγ/SOCS3 pathway in astilbin-downregulated CD4^+^ T cells (Figure 2K).

### PPARγ/Phosphatase and Tensin Homolog Pathway Involved in Astilbin-Treated CD4^+^ T Cells

**FIGURE 3:**
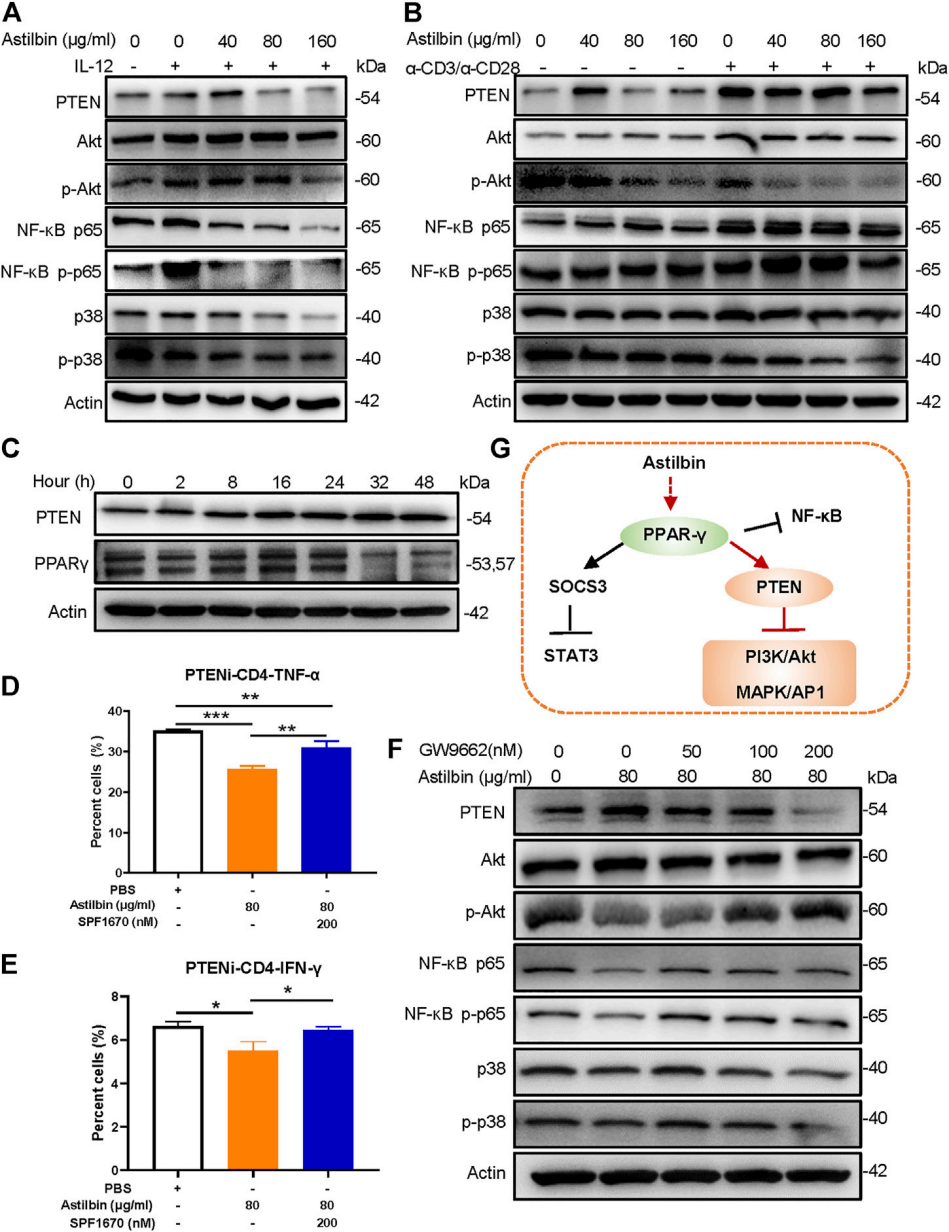
PPARγ/PTEN pathway in astilbin-treated CD4^+^ T cells. Effects of astilbin on the expression of PTEN, Akt/p-Akt, NF-κB p65/NF-κB p-p65, and p38/p-p38 of unstimulated, IL-12- **(A)**, or α-CD3/α-CD28− **(B)** stimulated CD4^+^ T cells by Western blot analysis. **(C)** Effects of astilbin (80 μg/ml) on PTEN and PPARγ of CD4^+^ T cells at different time points. Effects of PTEN inhibitor (SF1670) on TNF-α **(D)** and IFN-γ **(E)** production in astilbin-treated CD4^+^ T cells. Mean ± SD; *n* = 3. **(F)** Effects of GW9662 on PTEN, Akt/p-Akt, NF-κB p65/NF-κB p-p65, and p38/p-p38 of astilbin-treated CD4^+^ T cells. **(G)** Diagram of PPAR/PTEN pathway on CD4^+^T cells by astilbin. Each experiment was conducted at least three times. The inhibitor of PTEN, SF1670 is abbreviated as PTENi. *p* values (*p ≤ 0.05; **p ≤ 0.01; ***p ≤ 0.001) determined by one-way ANOVA **(D,E)**.

Given that astilbin conducts anti-inflammatory activity *via* downregulating the phosphorylation of PI3K/Akt, NF-κB, and p38 MAPK signaling pathways, some molecules with diphosphatase activity might be stimulated by astilbin. Both phosphatase and tensin homolog (PTEN) was a phosphatase that efficiently inhibits the activation of PI3K/Akt, NF-κB, and p38 MAPK (Worby and Dixon, 2014). Through astilbin treatment, PTEN is remarkably induced in physiological CD4^+^ T cells and IL-12-stimulated CD4^+^ T cells. Given that PTEN is more increased in CD4^+^ T cells by the stimulation of α-CD3/α-CD28, the induction of PTEN by astilbin may be sheltered in α-CD3/α-CD28-stimulated CD4^+^ T cells. Correspondingly, phosphorylated Akt, NF-κB p65, and p38 decreased in activated CD4^+^T cells treated by astilbin (Figures 3A,B; Supplementary Figures S3A,B). In addition, 2-h astilbin treatment increased PTEN expression, and the increased expression lasted for 48 h. Similarly, induced PPARγ expression was observed after 2–24-h stimulation (Figure 3C; Supplementary Figure S3C).

When astilbin-treated CD4^+^ T cells were co-cultured with a PTEN inhibitor (SF1670), TNF-α (Figure 3D) production was partially reversed (Supplementary Figure S3D), and IFN-γ (Figure 3E) production was restored, confirming that decreased cytokine secretion of CD4^+^ T cells is induced by astilbin dependent on the induction of PTEN. On the other hand, incomplete restoration of TNF-α indicated that PTEN was only one of the signal molecules involved in astilbin-induced downregulation. Given that PTEN expression is transactivated by PPARγ (Worby and Dixon, 2014), we determined whether the inhibition of PPARγ affected PTEN and its downstream signaling molecules. As shown in Figure 3F, when CD4^+^ T cells were cotreated by astilbin and GW9662, increased PTEN expression was blocked, and the decreased expression of phosphorylated Akt, NF-κB p65, and p38 was recovered (Supplementary Figure S3E). Taken together, astilbin promoted the PPARγ/PTEN pathway to downregulate TNF-α and IFN-γ production of CD4^+^ T cells (Figure 3G).

### Involvement of the PPARγ/AMPK Axis in Astilbin-Treated CD4^+^ T Cells

**FIGURE 4:**
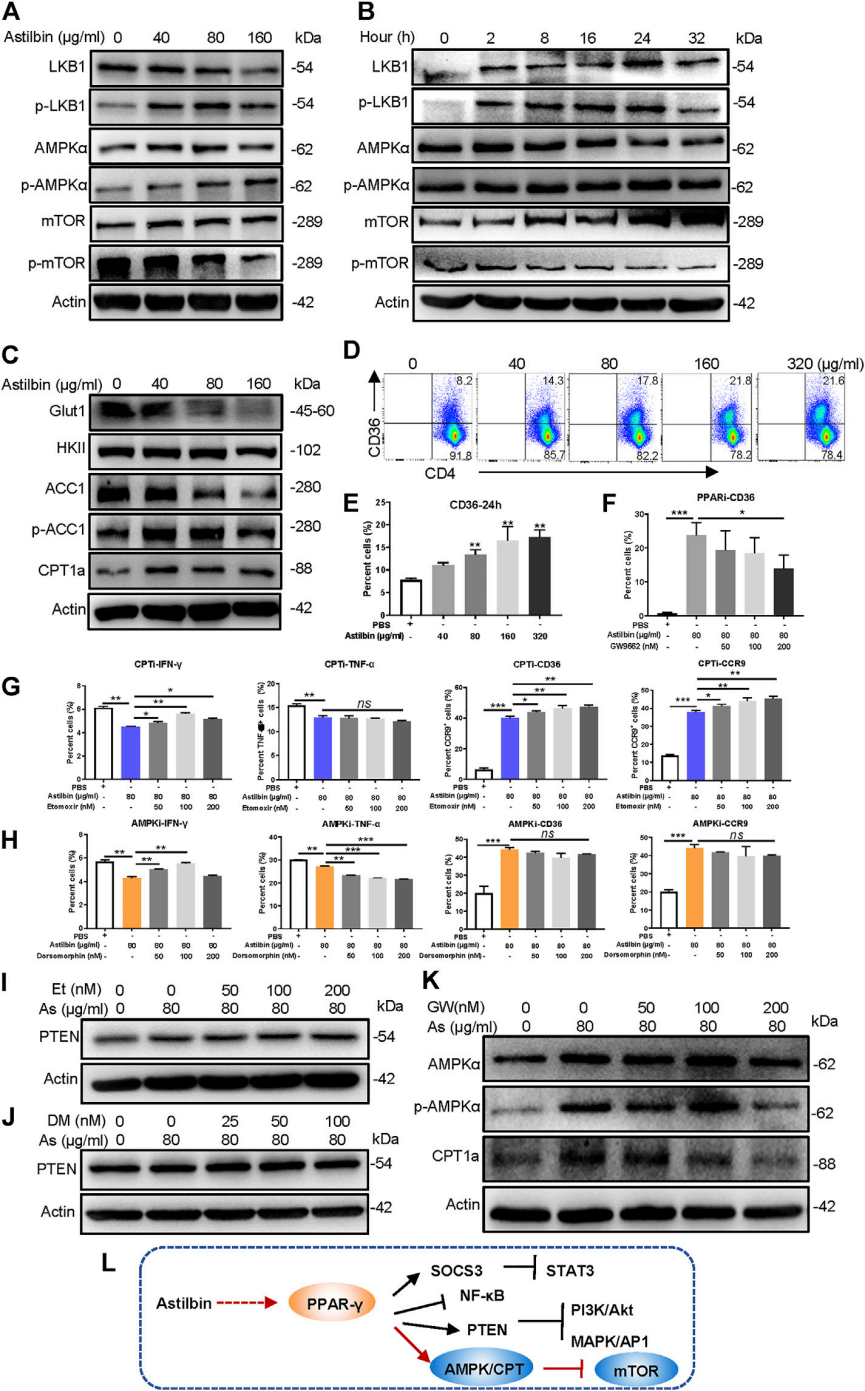
PPARγ/AMPK pathway in astilbin-treated CD4^+^ T cells. Variations of LKB1/p-LKB1, AMPK/p-AMPK, and mTOR/p-mTOR in CD4^+^ T cells treated with various doses of astilbin **(A)** or at different time points **(B)**. **(C)** Variations of proteins involved in glucose and lipid metabolism. CD36 of CD4^+^ T cells affected by astilbin **(D, E)**. Mean ± SD; *n* = 3. **(F)** Effects of PPARγ inhibition on CD36 expression. Mean ± SD; *n* = 3. Effects of CPT inhibition (etomoxir) **(G)** or AMPK inhibition (dorsomorphin) **(H)** on IFN-γ, TNF-α, CD36, and CCR9 of astilbin-treated CD4^+^ T cells. Mean ± SD; *n* = 3. Effects of etomoxir **(I)** or dorsomorphin **(J)** on PTEN expression of astilbin-treated CD4^+^ T cells. **(K)** Effects of PPARγ inhibition on AMPK and CPT1a expression of astilbin-treated CD4^+^ T cells. **(L)** Diagram of PPAR/PTEN pathway on CD4^+^ T cells by astilbin. Each experiment was performed at least three times. The inhibitor of CPT, etomoxir, is abbreviated as CPTi. The inhibitor of AMPK, dorsomorphin, is abbreviated as AMPKi. *p* values (*p ≤ 0.05; **p ≤ 0.01; ***p ≤ 0.001; ns, no significant difference) determined by one-way ANOVA **(E–H)**.

Astilbin decreases lipid accumulation in high-fat-diet-induced obese mice (Perez-Najera et al., 2018) and inhibits proliferation and improves differentiation in HaCaT keratinocytes *via* activating AMPK (C. Zhang et al., 2017). The LKB1/AMPK/mTOR signaling pathway was also checked in astilbin-treated CD4^+^ T cells. Although no substantial changes of total LKB1, AMPK, and mTOR level were observed, phosphorylated LKB1 and AMPK were increased, and phosphorylated mTOR was decreased in CD4^+^ T cells (Figure 4A; Supplementary Figure S4A). Moreover, the increased expression of phosphorylated LKB1 and AMPK was observed given that CD4^+^ T cells were treated with astilbin for 2 h, accompanied by the decreased activation of mTOR (Figure 4B; Supplementary Figure S4B).

Given the metabolic role regulated by AMPK, key molecules associated with glycolipid metabolism in CD4^+^ T cells were further analyzed. Through astilbin treatment, glucose transporter of T cells (Glut1) decreased with no obvious changes of HK2, indicating decreased glucose intake. Meanwhile, ACC1 (a key enzyme in fatty acid synthesis) was decreased, while CPT1a and pACC1 representing fatty acid oxidation (FAO) were increased (Figure 4C; Supplementary Figure S4C). The CD36 receptor for the intake of fatty acid was also increased in a dose-dependent manner by astilbin treatment for 24 (Figures 4D,E) or 36 h (Supplementary Figure S4D). These data demonstrated that astilbin treatment induced metabolic reprogramming of CD4^+^ T cells, namely, the upregulation of lipid oxidation and downregulation of lipid synthesis and glucose catabolism.

PPARγ activation can transactivate CD36 expression (Marechal et al., 2018). When CD4^+^ T cells were cotreated with astilbin and GW9662, the upregulation of CD36 was remarkably inhibited (Figure 4F; Supplementary Figure S4E). Next, we determined whether decreased CPT1a and p-AMPK exerted inhibitory effects on CD4^+^ T cell function. When a CPT1a inhibitor (etomoxir) was added, IFN-γ production could be partially increased, but TNF-α downregulation could not be reversed at all. On the contrary, etomoxir synergized with astilbin to promote CD36 and CCR9 expression (Figure 4G; Supplementary Figure S4F). Simultaneously, etomoxir had no effects on PTEN expression (Figure 4I; Supplementary Figure S4H). Thus, the increased CPT1a expression in CD4^+^ T cells was only a stimulatory effect induced by astilbin, not a cause for the downregulation of CD4^+^ T cell function.

Unexpectedly, when an inhibitor of AMPK (dorsomorphin) was expected to block astilbin-induced activation of AMPK, decreased IFN-γ production could be reversed, whereas TNF-α production was more decreased (Figure 4H; Supplementary Figure S4G). Meanwhile, treatment with dorsomorphin had no effects on the increased expression of CD36 and CCR9 induced by astilbin (Figure 4H; Supplementary Figure S4G). Dorsomorphin even synergized astilbin to express PTEN (Figure 4J; Supplementary Figure S4I), which could explain the deeper inhibition of TNF-α production by cotreatment of astilbin and dorsomorphin. It could be inferred that although the expression of TNF-α, IFN-γ, CCR9, and CCR6 of CD4^+^ T cells could be regulated by metabolic intervention, functional changes are very different when using different small molecules with metabolic modulatory activity. Given that the CD36 and CCR9 expression of astilbin-treated CD4^+^T cells was dependent on PPARγ (Figures 2I, 4F), the expression of p-AMPK and CPT1a was inhibited in CD4^+^ T cells by the cotreatment of GW9662 (Figure 4K; Supplementary Figure S4J). Collectively, astilbin stimulated the PPARγ/AMPK axis to induce metabolic reprogramming and downregulate the activities of CD4^+^ T cells (Figure 4L).

### Differential Expression Genes of CD4^+^ T Cells by Astilbin Treatment

**FIGURE 5:**
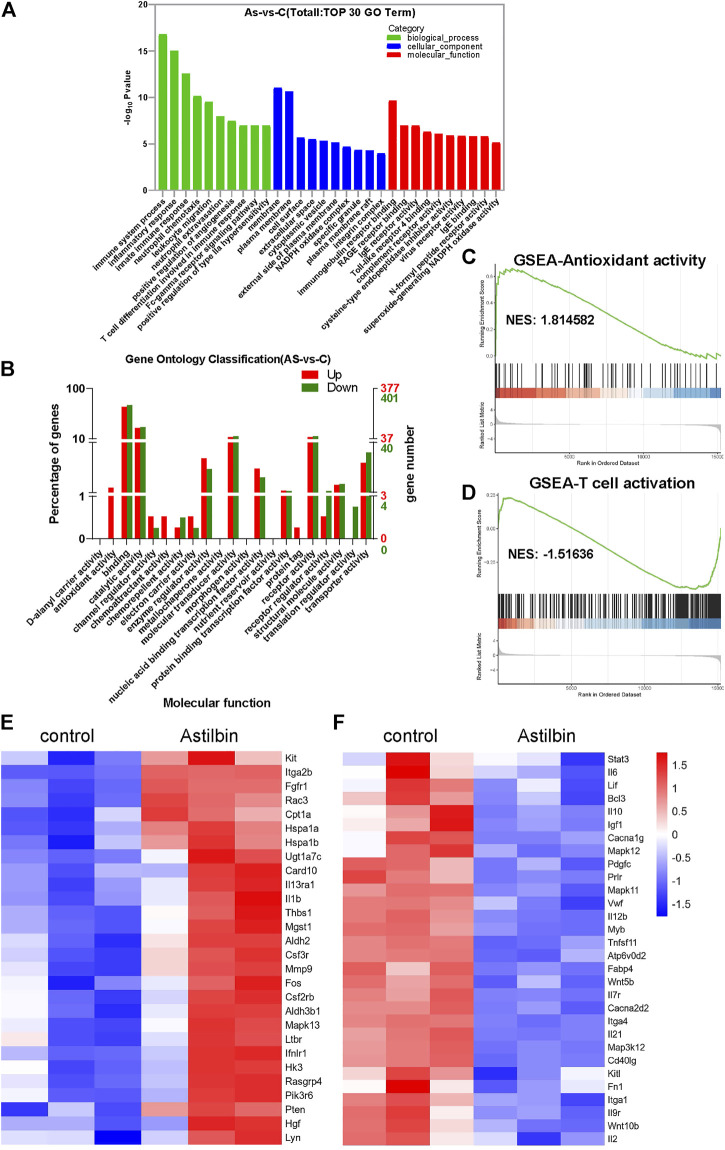
Transcriptome analysis of astilbin-treated CD4^+^ T cell functions. **(A)** Top 10 changed genes in biological process, cellular component, and molecular function. **(B)** Numbers of differentially expressed genes in molecular function by gene ontology analysis. GSEA of signaling molecules involved in antioxidant activity **(C)** and T cell activation **(D)**. Heatmap of upregulated **(E)** or downregulated **(F)** genes involved in immune function.

The transcriptome profiles of CD4^+^ T cells were further compared before and after astilbin treatment. The heatmap of mRNA sequencing results is displayed in Supplementary Figure S5A. Based on KEGG pathway analysis, the top three pathways were in the immune system (108 genes), signal transduction (104 genes), and signaling molecules and interaction (76 genes) (Supplementary Figure S5B). The most changed biological processes were immune system process, inflammatory process, and leukocyte migration by gene ontology analysis. Of note, the NADPH oxidase complex and superoxide-generating NADPH oxidase activity were among the top changed pathways of cellular component and molecular function, respectively (Figures 5A,C).

When the numbers of upregulated or downregulated genes associated with molecular function were checked, multiple genes were upregulated, but almost no genes related to the functions of antioxidant activity and chemoattraction were downregulated (Figure 5B). As expected, the JAK-STAT3, MAPK, and TNF signaling pathways which were associated with T cell activation, showed substantial changes (Figure 5D; Supplementary Table S1).

In addition, transcriptions of *PTEN*, *CPT1a*, and *microsomal glutathione S transferase 1* with antioxidase activity were upregulated in astilbin-treated CD4^+^ T cells (Figure 5E), while those of *Tnfsf11*, *Mapk11*, *IL-12*, *Mapk12*, *IL-12b*, *IL-2*, *STAT3*, and *IL-6* were decreased (Figure 5F). These results confirmed the downregulated expression of genes involved in inflammation and indicated the upregulation of genes with antioxidase activity in astilbin-treated CD4^+^ T cells.

### Astilbin Induced Cytoplasmic Reactive Oxidative Species Production to Activate PPARγ

**FIGURE 6:**
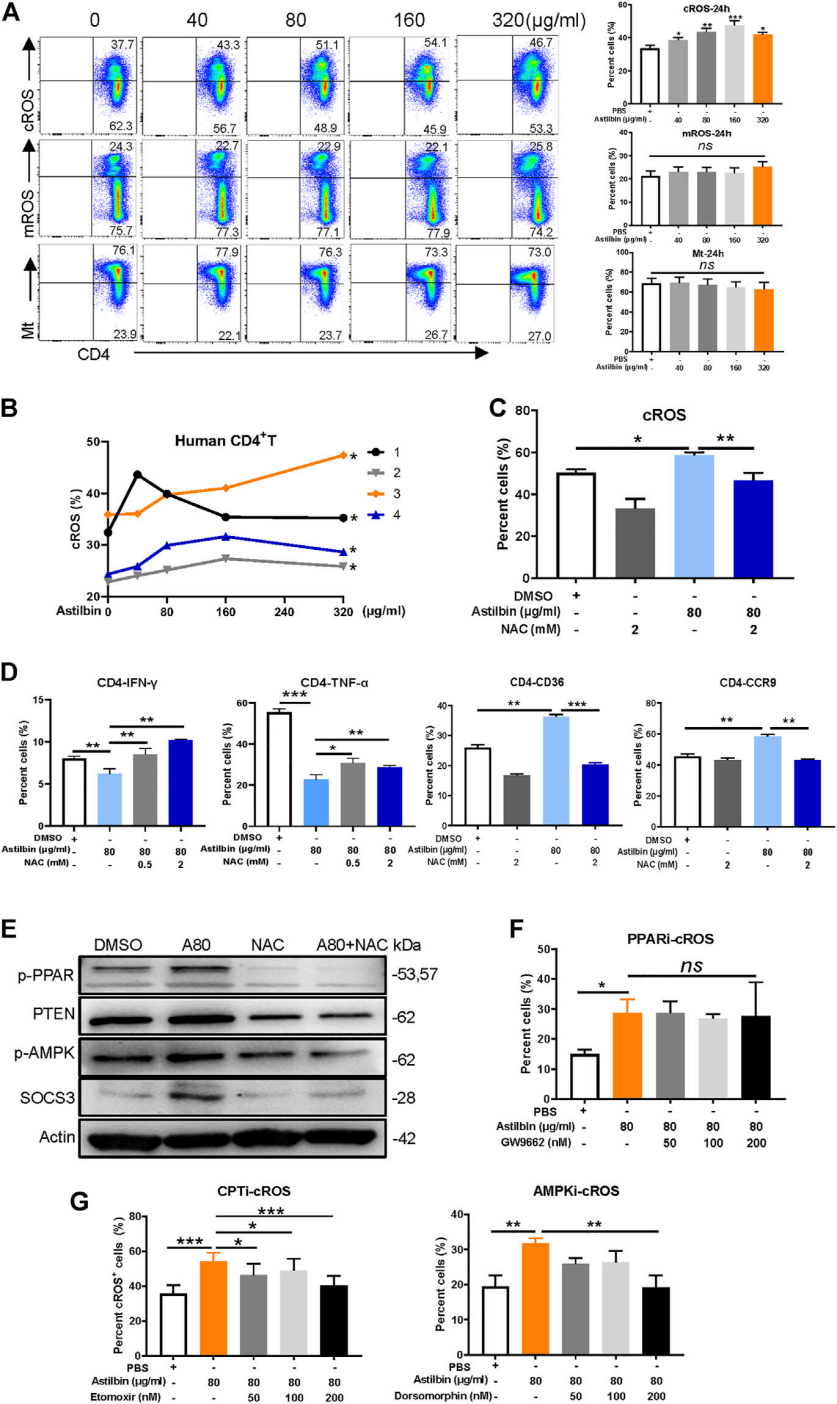
cROS production induced by astilbin in CD4^+^ T cells. **(A)** Variations of cytoplasmic ROS, mROS, and mitochondrial weight in murine CD4^+^ T cells treated with astilbin for 24 h. Mean ± SD; *n* = 3. **(B)** Variations of cROS in human CD4^+^ T cells treated with astilbin. Mean ± SEM; *n* = 4. **(C)** Depletion of ROS by NAC (2 mM). Mean ± SD; *n* = 3. **(D)** Effects of ROS depletion on IFN-γ, TNF-α, CD36, and CCR9 of astilbin-treated CD4^+^ T cells. Mean ± SD; *n* = 3. **(E)** Effects of ROS depletion on p-PPARγ, PTEN, p-AMPK, and SOCS3 of astilbin-treated CD4^+^ T cells. Effects of PPARγ inhibition **(F)** and CPT or AMPK inhibition **(G)** on cellular ROS production of astilbin-treated CD4^+^ T cells. Mean ± SD; *n* = 3. All experiments were conducted at least three times. *p* values (*p ≤ 0.05; **p ≤ 0.01; ***p ≤ 0.001; ns, no significant difference) determined by one-way ANOVA **(A,C,D,F,G)** and two-way ANOVA **(B)**.

Given that the transcriptions of NADPH oxidase complex and enzymes with antioxidant activity were increased in astilbin-treated CD4^+^ T cells, cytoplasmic and mROS of CD4^+^ T cells were measured after astilbin treatment. cROS were increased at astilbin doses of 40 and 80 μg/ml and peaked at 160 μg/ml (Figure 6A) after 24-h treatment. cROS also displayed similar patterns of variation after 36-h treatment (Supplementary Figure S6A). Unexpectedly, no obvious changes of mROS and mitochondrial weight were observed after 24-h treatment (Figure 6A); even mildly decreased mROS levels were observed after 36-h treatment (Supplementary Figure S6A). Human CD4^+^ T cells of peripheral blood from four healthy individuals also showed increased cROS production after astilbin treatment (Figure 6B).

Furthermore, Cellular ROS in astilbin-treated CD4^+^ T cells could be depleted by NAC (2 mM) (Figure 6C; Supplementary Figure S6B). When astilbin-treated CD4^+^ T cells were treated with NAC, decreased IFN-γ production and increased CCR9 and CD36 expression were completely restored (Figure 6D; Supplementary Figure S6C,D). PPAR expression is strongly induced and activated by ROS-oxidized lipids (Marion-Letellier et al., 2016). When NAC was added into CD4^+^ T cells, astilbin-increased expression of p-PPARγ, PTEN, p-AMPK, and SOCS3 was completely reversed (Figure 6E; Supplementary Figure S6E), confirming that PPARγ activation in astilbin-treated CD4^+^ T cells was dependent on cellular ROS production. Of note, TNF-α secretion was only partially reversed (Figure 6D; Supplementary Figure S6C,D), indicating that other factors were involved in this astilbin-induced downregulation.

Next, we determined whether inhibiting PPARγ, p-AMPK, or CPT1a would impact ROS production in astilbin-treated CD4^+^ T cells. When GW9662 was used to inhibit PPARγ activation, the increased ROS level induced by astilbin did not have any changes in CD4^+^ T cells (Figure 6F; Supplementary Figure S6F), indicating that the ROS production of CD4^+^ T cells was the upstream event of PPARγ activation as treated by astilbin. When FAO of CD4^+^ T cells was inhibited by CPT1a inhibitor (etomoxir) or AMPK inhibitor (dorsomorphin), ROS production induced by astilbin decreased (Figure 6G; Supplementary Figure S6F), indicating that the upregulated FAO induced by astilbin had a positive feedback for cellular ROS production. Taken together, astilbin induced cROS production in CD4^+^ T cells to activate the PPARγ signaling pathway.

### Astilbin Interacted With Cytochrome P450 1B1 to Stimulate Reactive Oxidative Species Production

**FIGURE 7:**
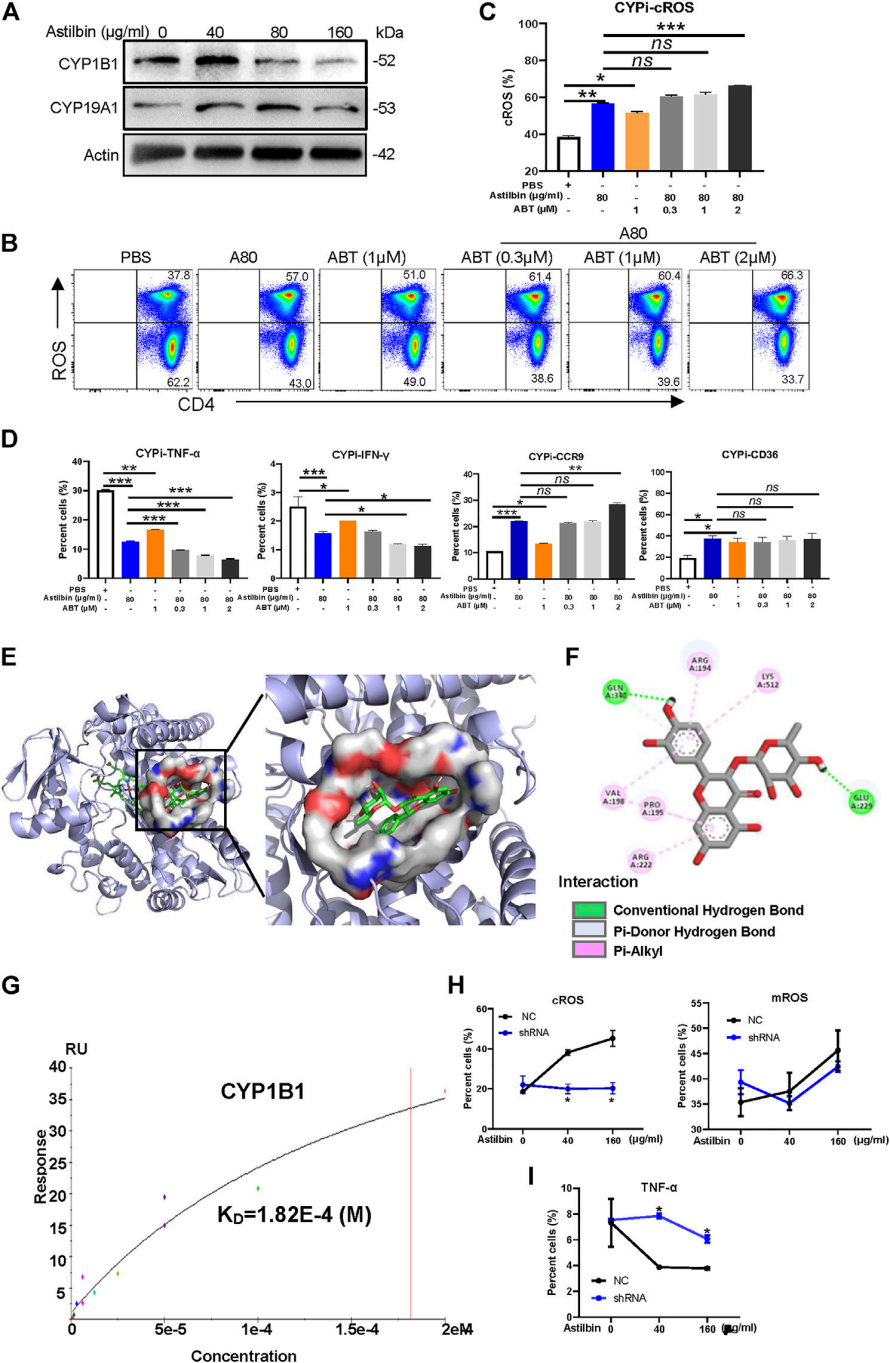
CYP1B1 provides a dock site for astilbin. **(A)** Variations of CYP1B1 and CYP19A1 in astilbin-treated CD4^+^ T cells. **(B,C)** Astilbin promotes cellular ROS production similar to ABT (a non-specific inhibitor of CYP). Mean ± SD; *n* = 3. **(D)** Effects of astilbin on IFN-γ, TNF-α, CD36, and CCR9 of CD4^+^ T cells similar to ABT. Mean ± SD; *n* = 3. **(E)** Three-dimensional model of the CYP1B1 active site with bound astilbin. **(F)** Binding of CYP1B1 with astilbin and the inset showing the principal interactive residues of astilbin at the CYP1B1 binding pocket. Green dashed lines represent hydrogen bonds. The hydrophobic interaction is depicted by pink dashed lines. **(G)** Real binding of CYP1B1 with astilbin confirmed by SPR assay. Production of cROS and mROS **(H)** and TNF-α **(I)** in CYP1B1 shRNA-transfected CD4^+^ T cells. Mean ± SEM; *n* = 3. All experiments were performed three times. The inhibitor of CYP, ABT, is abbreviated as CYPi. *p* values (*p ≤ 0.05; **p ≤ 0.01; ***p ≤ 0.001; ns, no significant difference) determined by one-way ANOVA **(C,D)** and two-way ANOVA **(H,I)**.

Finally, we aimed to investigate the molecular mechanisms of elevated ROS production in astilbin-treated CD4^+^ T cells. In general, cellular ROS is mainly produced by oxidative phosphorylation in mitochondria, cytochrome P450 (CYP) oxidases and endoplasmic reticulum oxidoreduction-1 in microsome, polyamine oxidases in cytoplasm, and cell membrane-coupled NADPH oxidases (Zhao et al., 2019). According to the integrated results predicted by the PharmMapper and UniProt databases, 33 candidate proteins of astilbin targets were determined, which are listed in Supplementary Table S2. Given that astilbin mainly induced cROS production of CD4^+^ T cells, we observed that two P450 enzymes (CYP1B1 and CYP19A1) were possibly docked by astilbin. Of note, the expression of CYP1B1 and CYP19A1 could be induced with astilbin (40 μg/ml), but downregulated (160 μg/ml) in CD4^+^ T cells (Figure 7A; Supplementary Figure S7A,B).

Next, ABT, a nonselective and irreversible inhibitor of CYP enzymes (Parrish et al., 2016), was used alone or in combination with astilbin to treat CD4^+^ T cells. As expected, ABT increased cROS production in CD4^+^ T cells. Compared with astilbin treatment (80 μg/ml) alone, a slightly lower level of cROS was observed in CD4^+^T cells treated by ABT (1 μM) alone. When low dose of ABT (0.3 or 1 μM) combined with astilbin (80 μg/ml) was used to treat CD4^+^ T cells, no enhancements of cROS were observed in the cytoplasm of CD4^+^ T cells compared with astilbin treatment (80 μg/ml) alone. However, cotreatment of high-dose ABT (2 μM) with astilbin (80 μg/ml) substantially increased cROS production (Figures 7B,C). This phenomenon indicated that astilbin promoted ROS production with similar molecular pattern to ABT.

As expected, ABT treatment (1 μM) alone reduced TNF-α and IFN-γ production to a lesser degree compared with the astilbin (80 μg/ml)-induced downregulation. In addition, there were obviously synergistic effects in TNF-α and IFN-γ production similar to cotreatment with astilbin (80 μg/ml) and ABT (1, 2 μM). Similar to astilbin, ABT (1 μM) alone increased CCR9 and CD36 expression. More increased CCR9 expression was only observed when ABT (2 μM) and astilbin (80 μg/ml) were both used. ABT and astilbin had no synergistic effects on CD36 expression, even if the ABT dose was 2 μM (Figure 7D; Supplementary Figure S7C). Thus, it was inferred that astilbin could use other molecular mechanisms to downregulate TNF-α and IFN-γ production besides ROS production; however, it induced CCR9 and CD36 expression dependent on ROS production.

The molecular docking study was conducted using AutoDock Vina 4.2.6. Binding affinity and presence of hydrogen or hydrophobic bonds between astilbin and CYP1B1 or CYP19A1 were predicted. Astilbin had higher binding affinity with CYP1B1 (−6.4 kcal/mol) than CYP19A1 (−3.3 kcal/mol). Then, we used computer-assisted modeling to further investigate whether astilbin interacted with CYP1B1 directly (Figure 7E). Three hydrogen bonds were formed in the astilbin–CYP1B1 complex of Glu229 and Gln340 residues (Figure 7F). In addition, six hydrophobic contacts contributed to the binding, namely, Pro195, Val198, Arg222, Arg194, Val198, and Lys512 (Supplementary Table S3). These interactions helped CYP1B1 and astilbin form stable complexes. Recombinant CYP1B1 and CYP19A1 proteins were used to detect the interaction with astilbin by SPR assay. CYP1B1 stably docked astilbin with a dissociation rate (K_D_) of 18.2 (μM) (Figure 7G), but CYP19A1 could not bind astilbin efficiently (data not shown). When CYP1B1 was specifically knocked down by shRNA, the increase of cROS in astilbin-treated CD4^+^T cells disappeared with no variations of mROS production (Figure 7H; Supplementary Figure S7D). The decreased CYP1B1 expression in shRNA-transfected CD4^+^ T cells was confirmed in Supplementary Figure S7E,F. Consequently, the decrease of TNF-α production in CD4^+^ T cells induced by astilbin was reversed after being transfected with CYP1B1 shRNA (Figure 7I; Supplementary Figure S7G). Taken together, we identified that astilbin directly binds CYP1B1 to promote ROS production for the decreased secretion of inflammatory cytokines in CD4^+^ T cells.

## Discussion

The effects and molecular mechanisms of astilbin on modulating effector CD4^+^ T cell functions were comprehensively analyzed in this study. Astilbin decreased TNF-α and IFN-γ production and increased CCR9 expression of CD4^+^ T cells, and played a protective role in the onset of type 1 diabetes. PPARγ activation was the upstream event induced by astilbin, leading to stimulate the downstream PTEN and SOCS3 expression and LKB1/AMPK activation. Correspondingly, signaling molecules in mediating the effector function of CD4^+^ T cells, such as STAT3, NF-κB, Akt, p38 MAPK, and mTOR, were downregulated. Transcriptome sequencing results confirmed the changes of signaling molecules involved in immune systems and inflammatory responses and indicated key variations of enzymes with oxidant or antioxidant activities. Finally, with the help of bioinformatics analysis and SPR assay, we identified that CYP1B1 had a docking pocket for astilbin and that after astilbin interacted with CYP1B1, cellular ROS would be sharply induced to downregulate CD4^+^ T cell functions, similar to the effects mediated by a nonspecific inhibitor of P450 (ABT). These results elucidated that CYP1B1 is one docking receptor used by astilbin to promote ROS production for the downregulation of effector CD4^+^T cell function.

CYP1B1 is a member of the CYP superfamily of enzymes localized in the endoplasmic reticulum. The CYP proteins are monooxygenases that catalyze many reactions involved in drug metabolism and synthesis of cholesterol, steroids, and other lipids. CYP1B1 catalyzes the hydroxylation of aryl compounds, thus generating more polar metabolites. About 30 flavonoid compounds have been demonstrated to inhibit CYP1B1 activity (Dutour and Poirier, 2017). Here, we found that astilbin was another inhibitor of CYP1B1. Compared with other CYP1B1 inhibitors derived from flavones, astilbin bound CYP1B1 less potently due to the relatively low affinity (K_D_ = 18.2 μM). Possibly, the lower binding affinity led to moderate but not high levels of ROS production in CD4^+^ T cells as treated by astilbin (∼80 μg/ml). The moderate or appropriate levels of ROS in cells could efficiently produce oxidized lipids to stimulate PPARγ activation, induce antioxidant expression, and avoid cell apoptosis induced by high levels of ROS in cells. Considering the IC_50_ (181 μg/ml), astilbin treatment at a dose of 40–80 μg/ml is safe and effective in the downregulation of CD4^+^ T cell activities.

**FIGURE 8:**
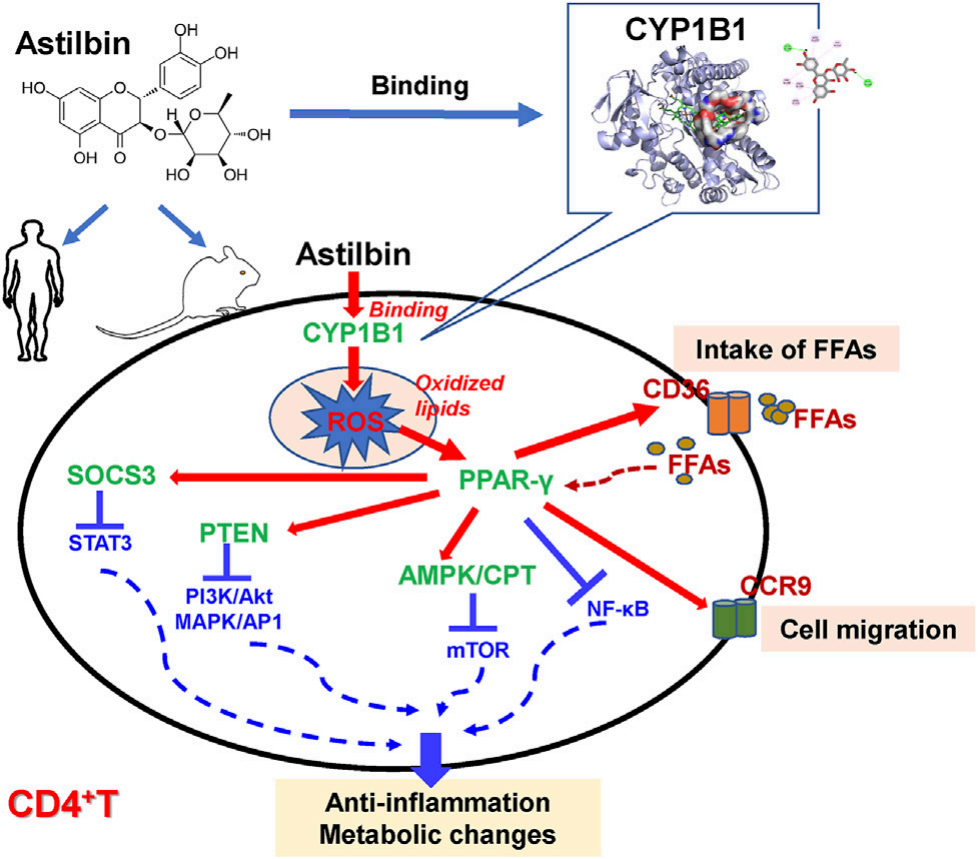
Diagram of astilbin-modulated functions of CD4^+^ T cells.

Inhibiting PAPRγ activation by GW9662 could almost restore the TNF-α, IFN-γ, and CCR9 expression of astilbin-treated CD4^+^ T cells. Moreover, GW9662 treatment restored the expression of SOCS3, PTEN, p-Akt, NF-κB pp65, pp38 MAPK, p-AMPK, and CPT1a. It could be concluded that PPARγ activation was a key molecular event in astilbin-induced downregulation of CD4^+^ T cells. Meanwhile, PPARγ directly transactivated the expression of CCR9 and CD36. The enhancement of CD36 would promote CD4^+^ T cells to take in free fatty acids (FFAs), and as a positive feedback, intracellular FFAs would promote more PPAR expression. The diagram of key molecular pathways involved in astilbin-treated CD4^+^ T cells is shown in Figure 8. Meanwhile, another antioxidant transcriptional factor, Nrf2, was also induced by astilbin treatment. However, astilbin still downregulated TNF-α production of *Nrf2* ^*−/−*^ CD4^+^T cells, demonstrating that Nrf2 did not play the key role in the process.

Astilbin was shown to promote the induction of regulatory NK1.1^−^ CD4^+^ NKG2D^+^ T cells and induce regulatory B cells if in combination with LPS dependent on the STAT3 pathway. In astilbin-induced NK1.1^−^ CD4^+^ NKG2D^+^ T cells, STAT3, and SOCS3 were upregulated (21). However, pSTAT3 (Tyr 705) was increased at a dose of 40 μg/ml but decreased at 160 μg/ml when B cells were treated with astilbin alone. However, no changes in SOCS3 expression were observed. Considering the increased SOCS3 and decreased pSTAT3 expression in astilbin-treated effector CD4^+^ T cells, it was inferred that astilbin used distinct molecular mechanisms to modulate the functions of different cells. Meanwhile, our results confirmed that STAT3 activation in different cell types impacts different effects on cell function.

PTEN expression was decreased by using the PPARγ inhibitor (GW9662) but was increased by the AMPK inhibitor (dorsomorphin) in astilbin-treated CD4^+^T cells, indicating that PTEN expression was regulated by multiple factors. Although the mROS and mitochondrial weight of CD4^+^ T cells treated with astilbin for 24 h showed no obvious changes, they decreased after 36-h treatment (Supplementary Figure S6), confirming the protective role of astilbin in cerebral ischemia/reperfusion injury by downregulating ROS-NLPR3 activation (Li et al., 2020). Mild decrease of TGF-β1 was also observed in astilbin-treated CD4^+^ T cells, which was possibly due to decreased STAT3 activation (Dai et al., 2021). The downregulation of TGF-β1 at the transcriptional level is also involved in renal damage in adenine-induced chronic renal failure rats mediated by astilbin-included Erhuang Formula (C. Y. Zhang C Yet al., 2017). Given the key role of TGF-β1 in fibrosis-associated diseases, this effect may be one mechanism in anti-fibrosis activity mediated by astilbin (Sun et al., 2021).

According to multiple spectroscopic coupled with molecular docking analysis, astilbin has strong binding ability to human CYP2D6 (Tao et al., 2021). Ultrahigh-performance liquid chromatography and triple quadrupole mass spectrometry have been used to confirm the interaction of human CYP3A4 and CYP2D6 with astilbin (Shi et al., 2021). In the present study, astilbin efficiently stimulated the ROS of human CD4^+^ T cells, indicating the inhibition of CYP3A4 and CYP2D6 by astilbin. However, whether astilbin can bind human CYP1B1 or mouse CYP3A4/CYP2D6 still needs further study. In addition, cotreatment of astilbin and the CYP non-selective inhibitor exerted synergistic effects on TNF-α and IFN-γ production in CD4^+^ T cells. We could not exclude the inhibitory effects mediated by other candidate targets such as STAT1 or MAPKp38 as predicted by bioinformatics (Supplementary Table S2).

CD4^+^ T cells have been regarded as playing a key role in the pathogenesis of T1D (Haskins and Cooke, 2011). The relevance of Th1 cells to T1D in humans has been confirmed by many studies on CD4^+^ T cells isolated from human patients. Studies have shown that reactive oxygen species (ROS) act as signaling molecules contributing to T cell fate and function (Chavez and Tse, 2021). Eliminating autoreactive T cells by targeting ROS production is a potential strategy to inhibit autoreactive T cell activation without compromising systemic immune function. Recently, more attention has been focused on various treatments to prevent the destructive activity of CD4^+^ T cells (Bediaga et al., 2022), as our study showed that astilbin can effectively inhibit the activity of CD4^+^ T cells in NOD mice and humans, providing a new idea for the treatment of T1D. However, it is still necessary to further explore how astilbin inhibits CD4^+^ T cells activity and whether it targets ROS production in T1D.

In conclusion, we demonstrated that murine CYP1B1 was one P450 receptor of astilbin in CD4^+^ T cells. The CYP1B1 inhibition by astilbin promoted ROS production and induced the expression of PPAR and antioxidant enzymes. PPAR activation led to the decreased expression of inflammatory cytokines and increased expression of CD36 and CCR9 in CD4^+^ T cells *via* the stimulation of PTEN, SOCS3, and AMPK pathways (Figure 8). Given that elevated levels of CYP1B1 were observed in multiple cancers (Li et al., 2017), astilbin could also exert anti-tumor activity *via* targeting CYP1B1 to induce apoptosis by producing high levels of ROS.

## Data Availability

The datasets presented in this study can be found in online repositories. The names of the repository/repositories and accession number(s) can be found below: https://www.ncbi.nlm.nih.gov/, PRJNA798859.
