# Supplementary figures and images for: Astilbin Activates the Reactive Oxidative Species/PPARγ Pathway to Suppress Effector CD4+ T Cell Activities via Direct Binding With Cytochrome P450 1B1
Source: Front Pharmacol. 2022 May 16;13:848957. doi: 10.3389/fphar.2022.848957 (PMC9150850; doi:10.3389/fphar.2022.848957)

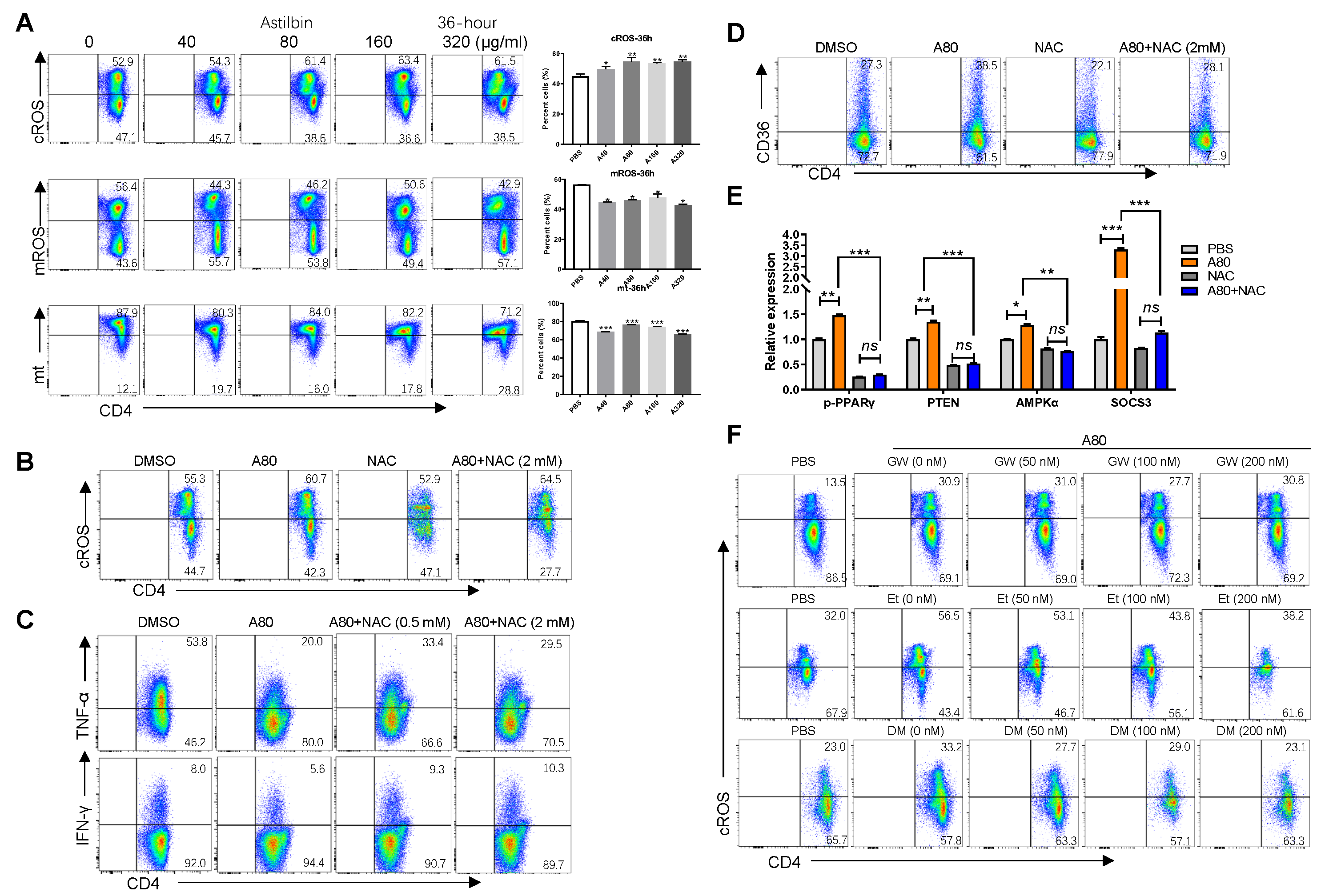

Supplement: Supplementary file 1 [file Image6.TIF]

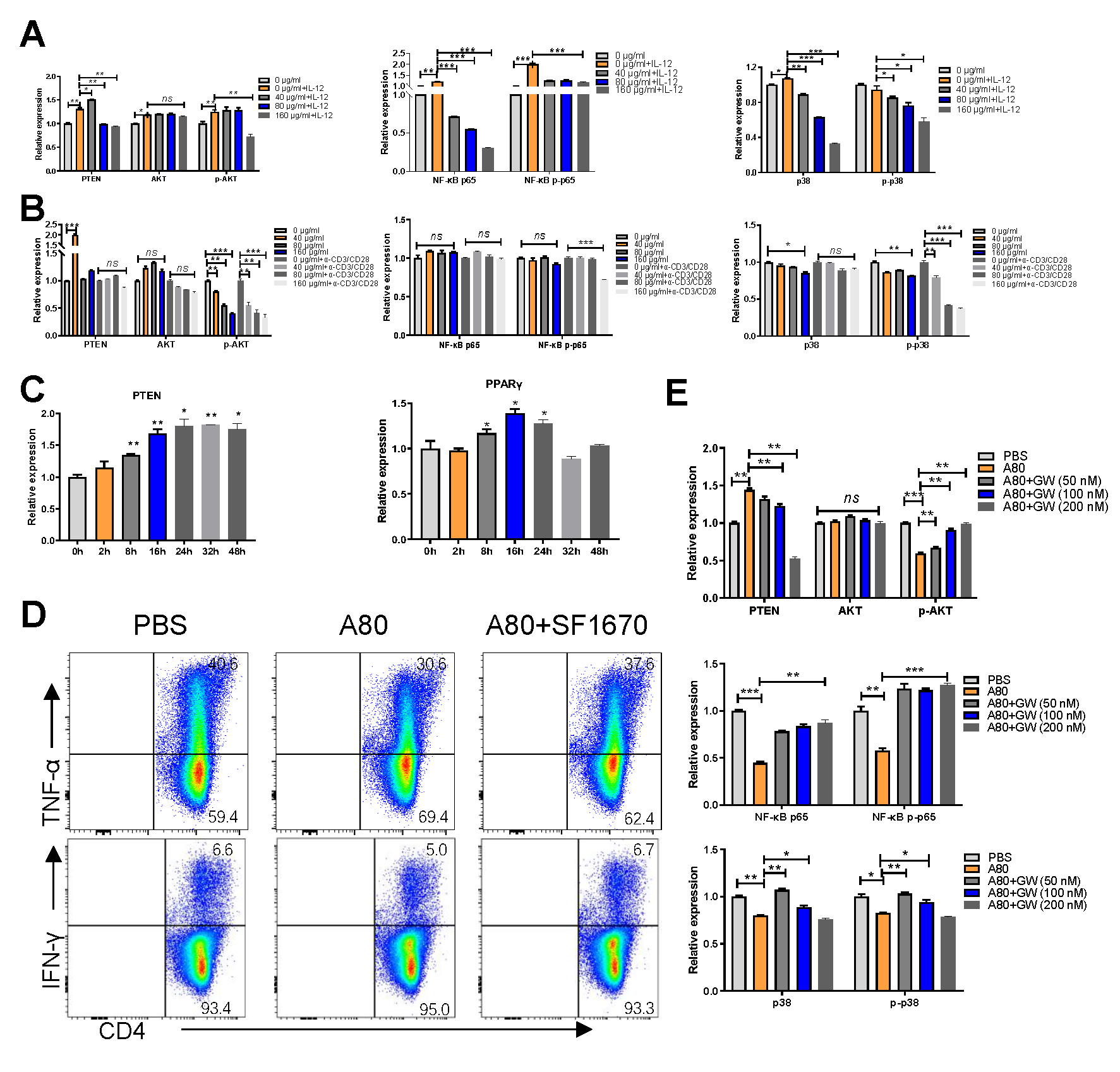

Supplement: Supplementary file 2 [file Image3.TIF]

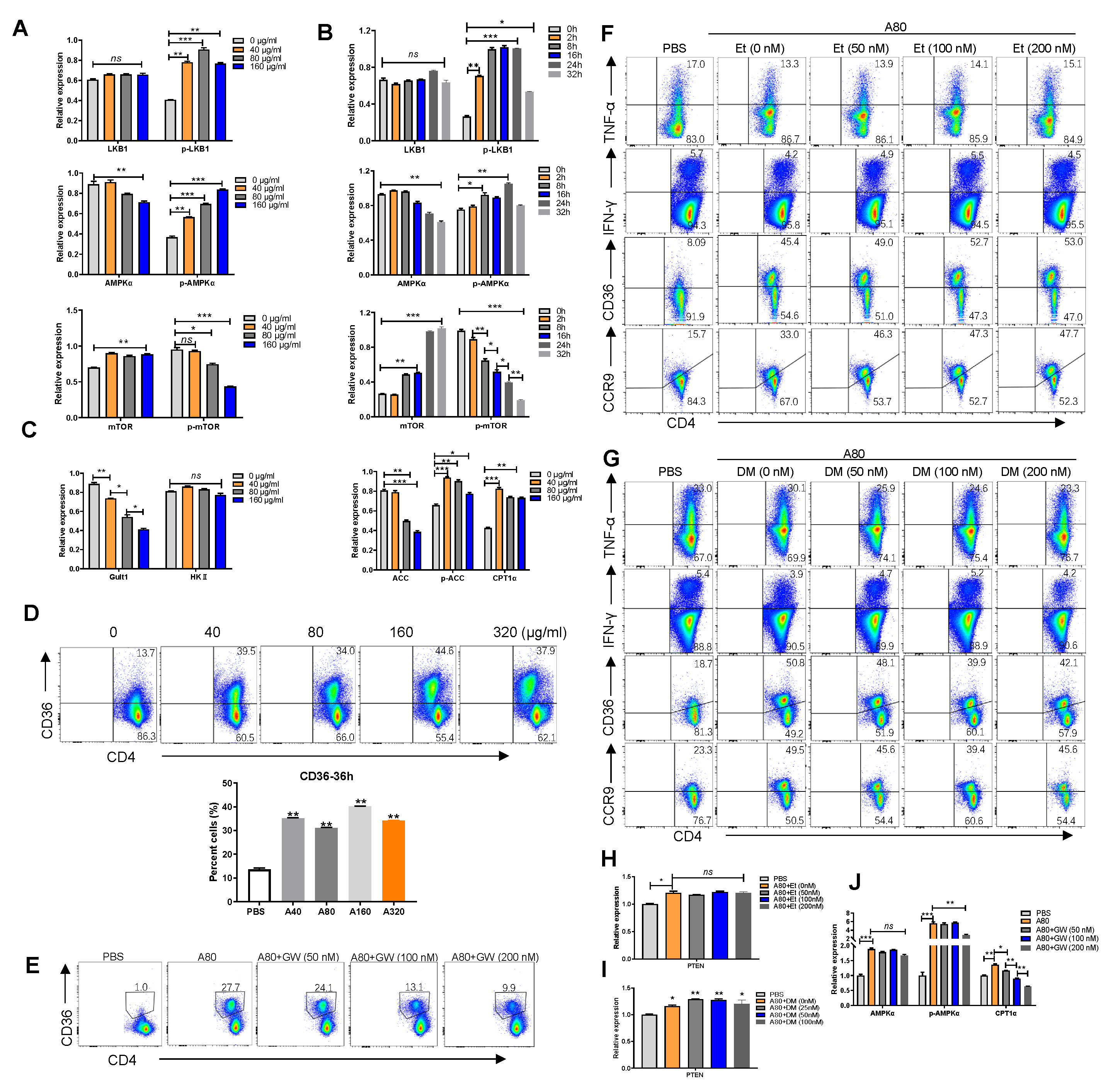

Supplement: Supplementary file 3 [file Image4.TIF]

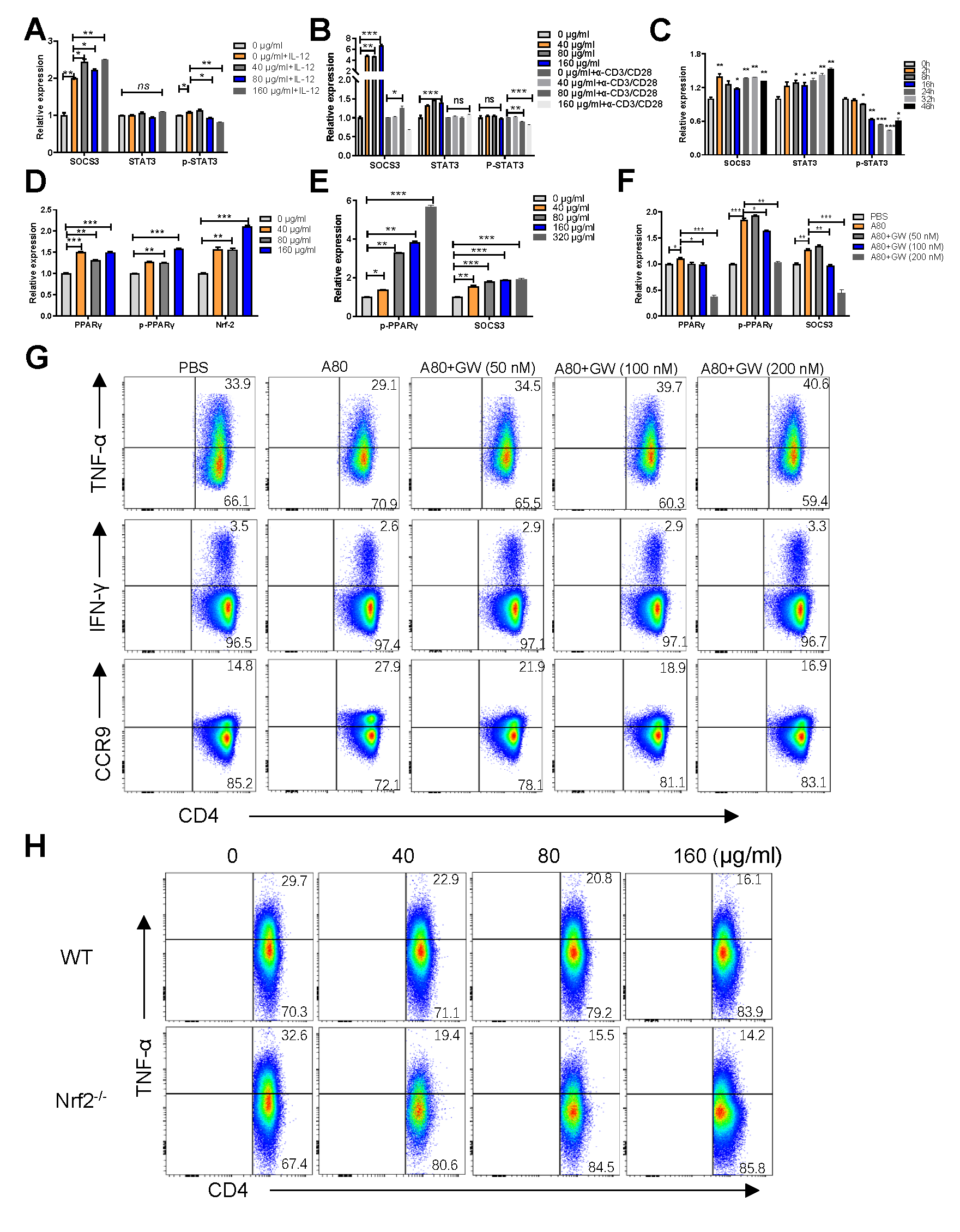

Supplement: Supplementary file 4 [file Image2.TIF]

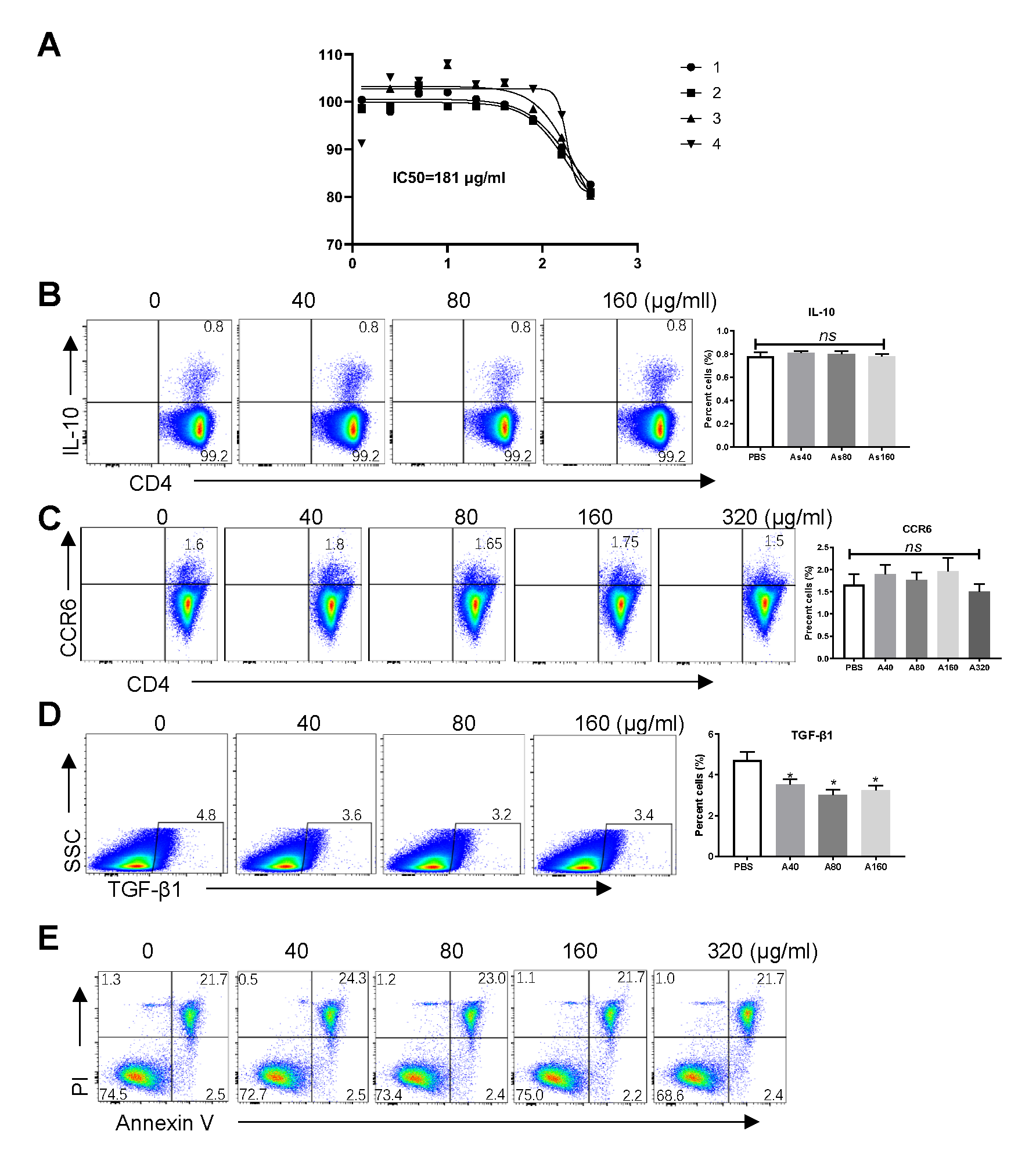

Supplement: Supplementary file 5 [file Image1.TIF]

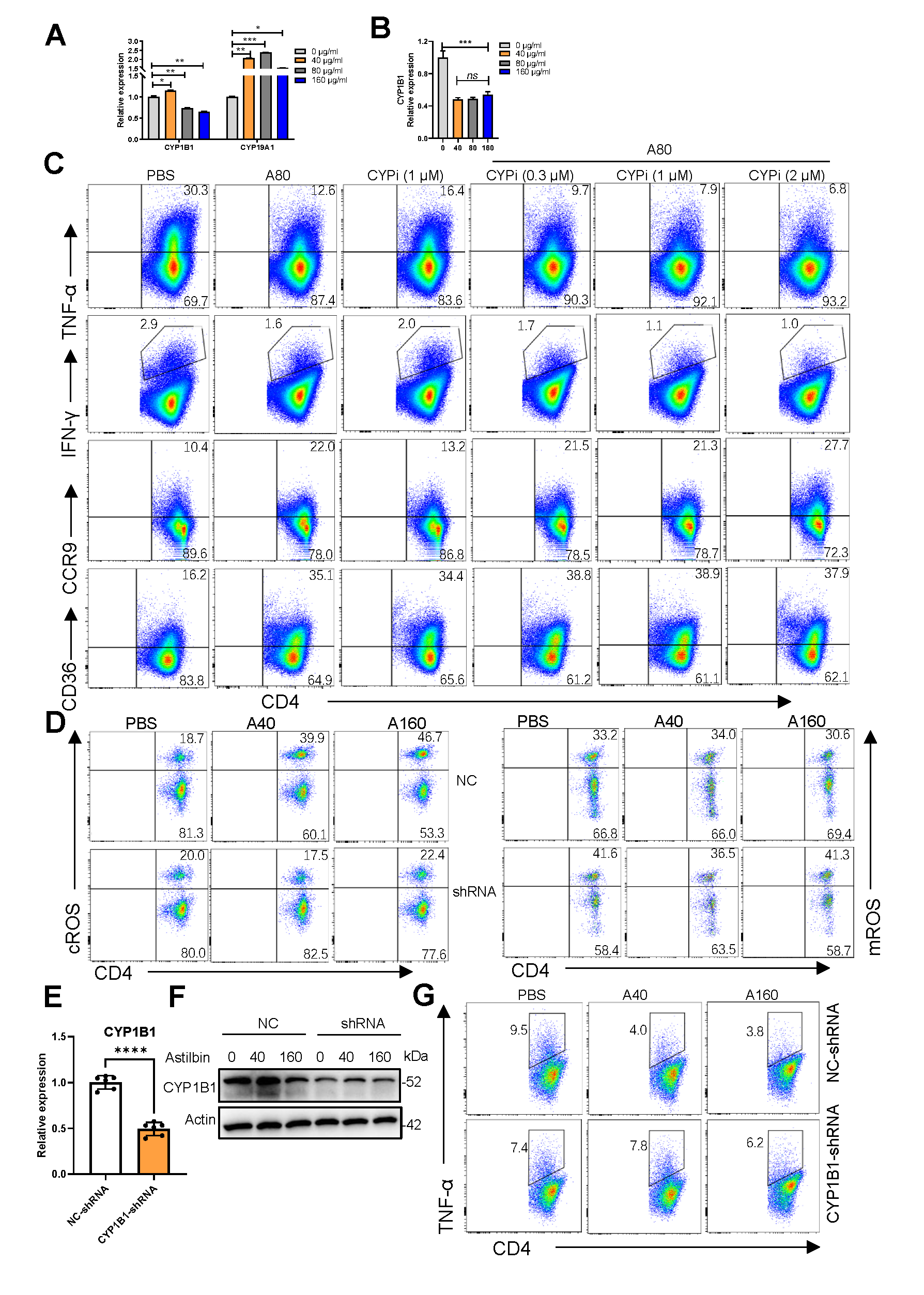

Supplement: Supplementary file 6 [file Image7.TIF]

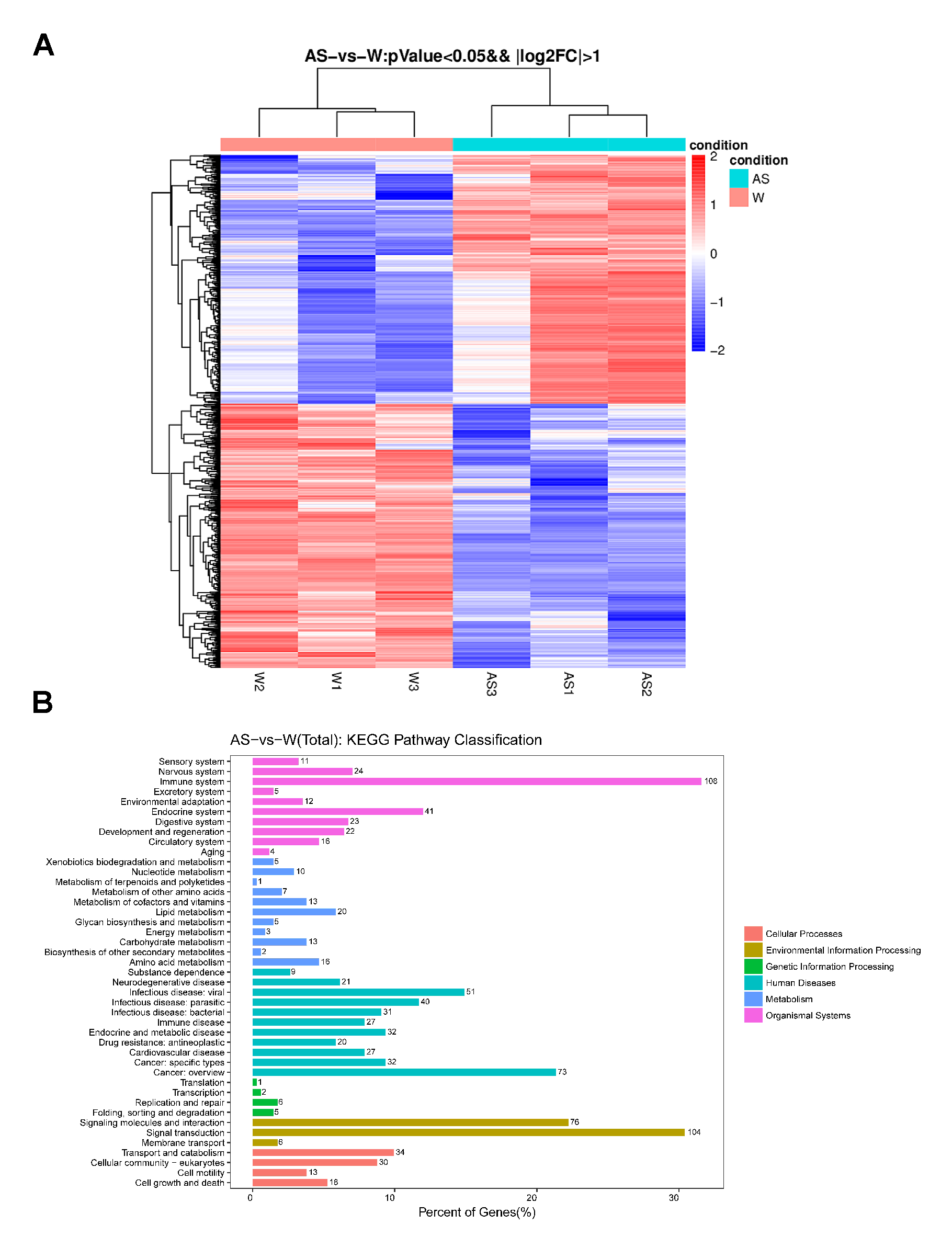

Supplement: Supplementary file 9 [file Image5.TIF]
