# Supplementary material for: Astilbin Activates the Reactive Oxidative Species/PPARγ Pathway to Suppress Effector CD4+ T Cell Activities via Direct Binding With Cytochrome P450 1B1
Source: Front Pharmacol. 2022 May 16;13:848957. doi: 10.3389/fphar.2022.848957 (PMC9150850; doi:10.3389/fphar.2022.848957)
Supplement: Supplementary file 7 [file DataSheet1.PDF]

Supplementary Material

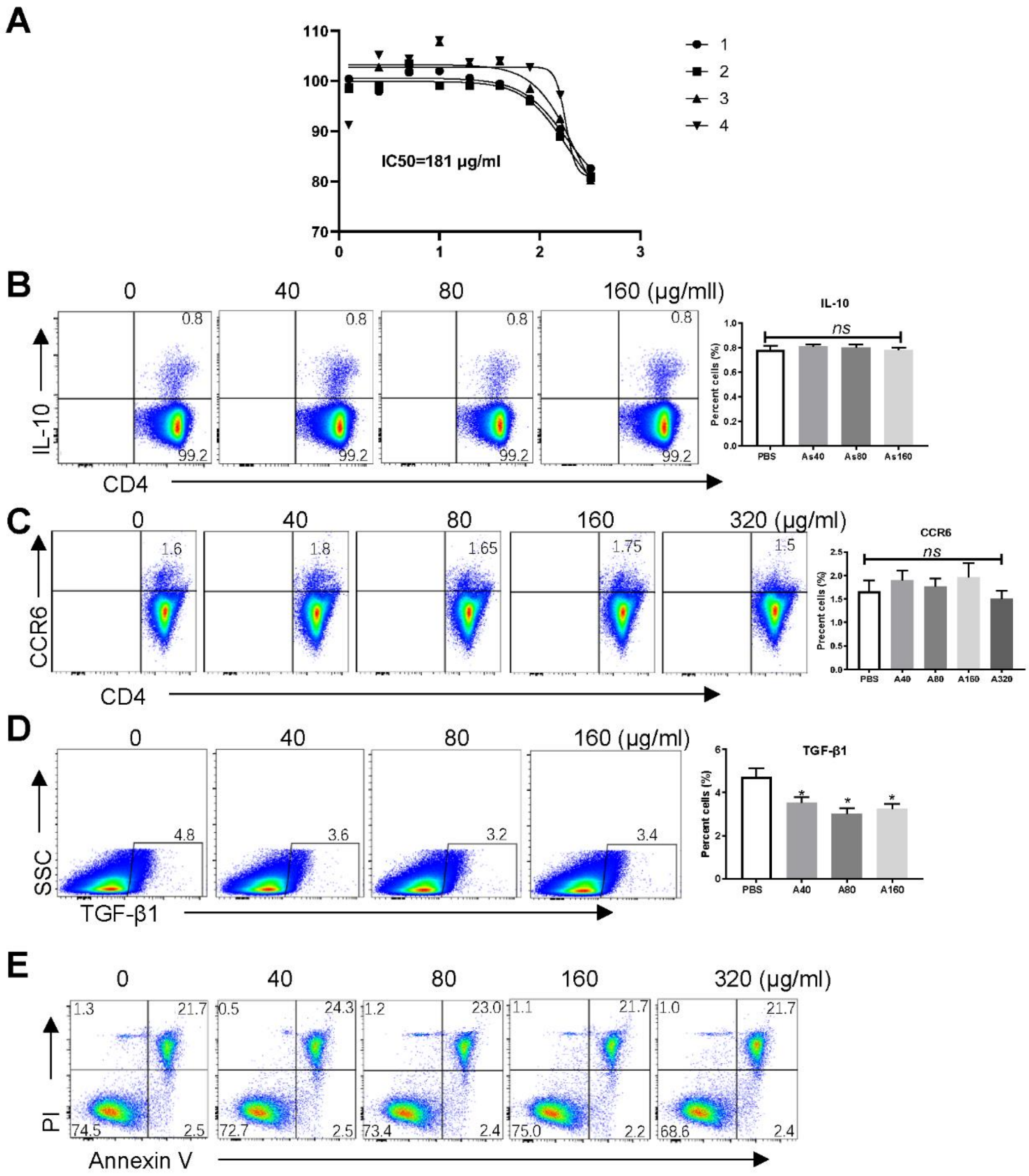

Supplementary Figure 1. (**A**), IC50 of astilbin on mouse CD4$^+$ T cells. (**B**) Gating strategies of IL-10 and ther gating strategies are similar. IL-10 (**B**), CCR6 (**C**) and TGF-β1 (**D**) production of CD4$^+$ T cells. Mean±SD; n=3. (**E**). Astilbin-induced apoptosis of CD4$^+$T cells detected by the staining of annexin V and PI. All experiments were performed three times. P values (**P* ≤ 0.05; ns, no significant difference) determined by one-way ANOVA (**B-D**).

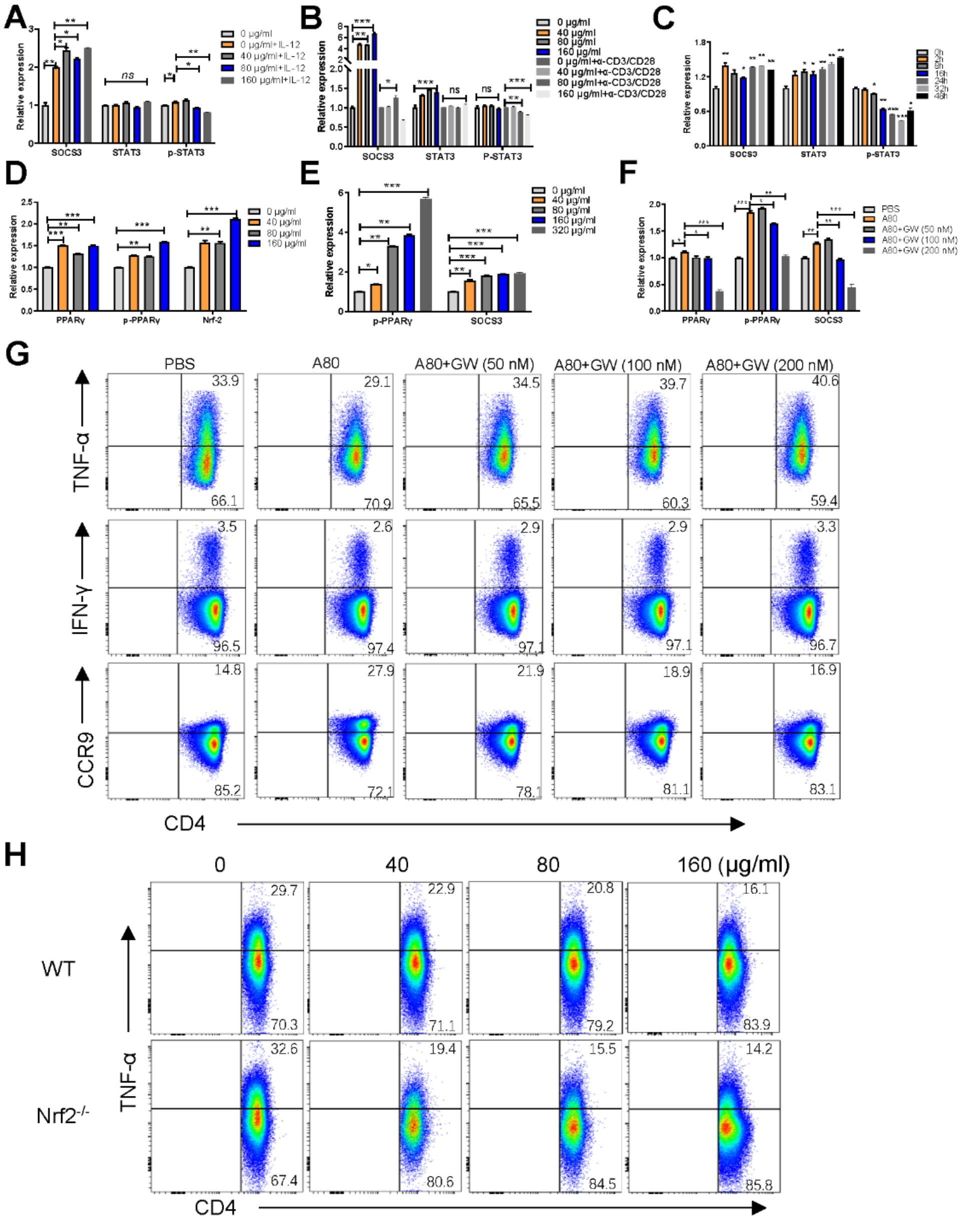

Supplementary Figure 2. Statistical analysis of expression levels of SOCS3, STAT3, p-STAT3 of astilbin-treated T cells by the IL-12 (**A**) and α-CD3/CD28 (**B**) stimulation. (**C**). Expression levels of SOCS3, STAT3, p-STAT3 in CD4$^+$ T cells by the treatment of astilbin (80 μg/ml) at different time courses. (**D**). Variations of PPARγ, p-PPARγ, and Nrf2. (**E**). Statistics of p-PPARγ and SOCS3 expression treated with astilbin. (**F**). Statistics of PPARγ inhibition on SOCS3 expression. (**G**). Representative results of PPARγ inhibition on TNF-α, IFN-γ, and CCR9 of astilbin-treated CD4$^+$ T cells. (**H**). Representative results of TNF-α in astilbin-treated CD4$^+$ T cells from *Nrf2*$^{-/-}$ mice. Mean±SD; n=3. All experiments were performed three times. P values (*$P \leq 0.05$; **$P \leq 0.01$; ***$P \leq 0.001$; ns, no significant difference) determined by one-way ANOVA (**A-F**).

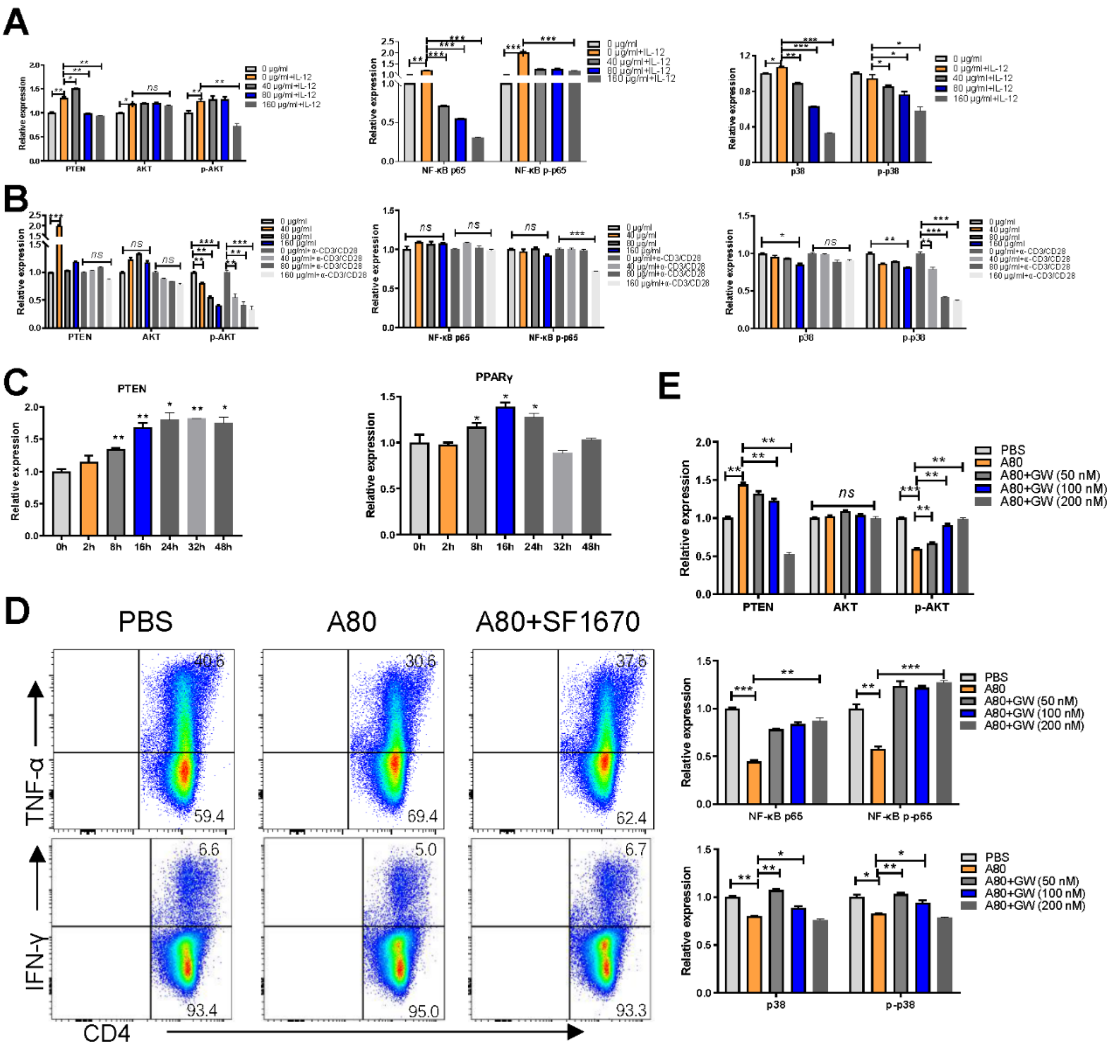

Supplementary Figure 3. Statistical analysis of expression levels of PTEN, AKT, p-AKT; NF-κB p65, NF-κB p-p65; and p38, p-p38 of astilbin-treated CD4$^+$ T cells by the IL-12 (**A**) or α-CD3/CD28 (**B**) stimulation. (**C**). Statistics of PTEN, PPARγ in 80 μg/ml astilbin-treated CD4$^+$ T cells at different time courses. (**D**). Representative results of TNF-α and IFN-γ in astilbin-treated CD4$^+$ T cells after PPAR inhibition. (**E**). Statistical analysis of expression levels of PTEN, AKT, p-AKT; NF-κB p65, NF-κB p-p65; and p38, p-p38 of astilbin-treated CD4$^+$ T cells after PPAR inhibition. Mean±SD; n=3. All experiments were performed three times. P values (*$P \leq 0.05$; **$P \leq 0.01$; ***$P \leq 0.001$; ns, no significant difference) determined by one-way ANOVA (**A-C,E**).

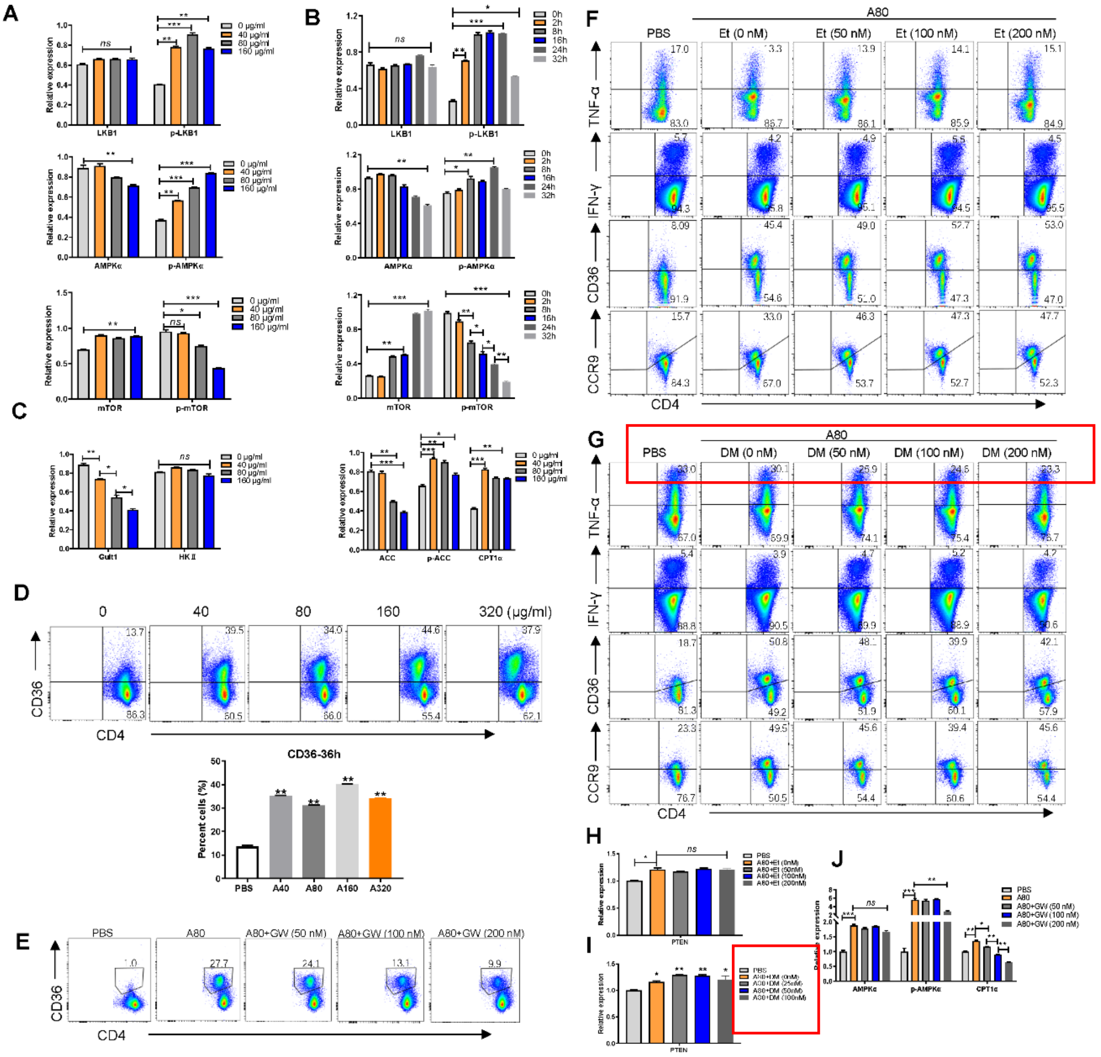

Supplementary Figure 4. Statistical analysis of expression levels of LKB1, p-LKB1; AMPKα, p-AMPKα; and mTOR, p-mTOR of astilbin-treated CD4$^+$ T cells (**A**) or at different time courses (**B**). Glut1, HK II, ACC, p-ACC, CPT1α (**C**) expression of astilbin-treated CD4$^+$ T cells. Effects of astilbin on CD36 expression by the astilbin treatment alone (**D**) or with PPAR inhibition (**E**). Representative results of TNF-α, IFN-γ, CD36 and CCR9 in astilbin-treated CD4$^+$ T cells after CPT1α (**F**) or (**G**) AMPKα inhibition. Effects of CPT1α (**H**) or (**I**) AMPKα inhibition on PTEN expression in astilbin-treated CD4$^+$ T cells. J. Effects of PPAR inhibition on AMPKα, p-AMPKα, and CPT1α expression in astilbin-treated CD4$^+$ T cells. Mean±SD; n=3. All experiments were performed three times. P values (*$P \leq 0.05$; **$P \leq 0.01$; ***$P \leq 0.001$; ns, no significant difference) determined by one-way ANOVA (**A-D,H-J**).

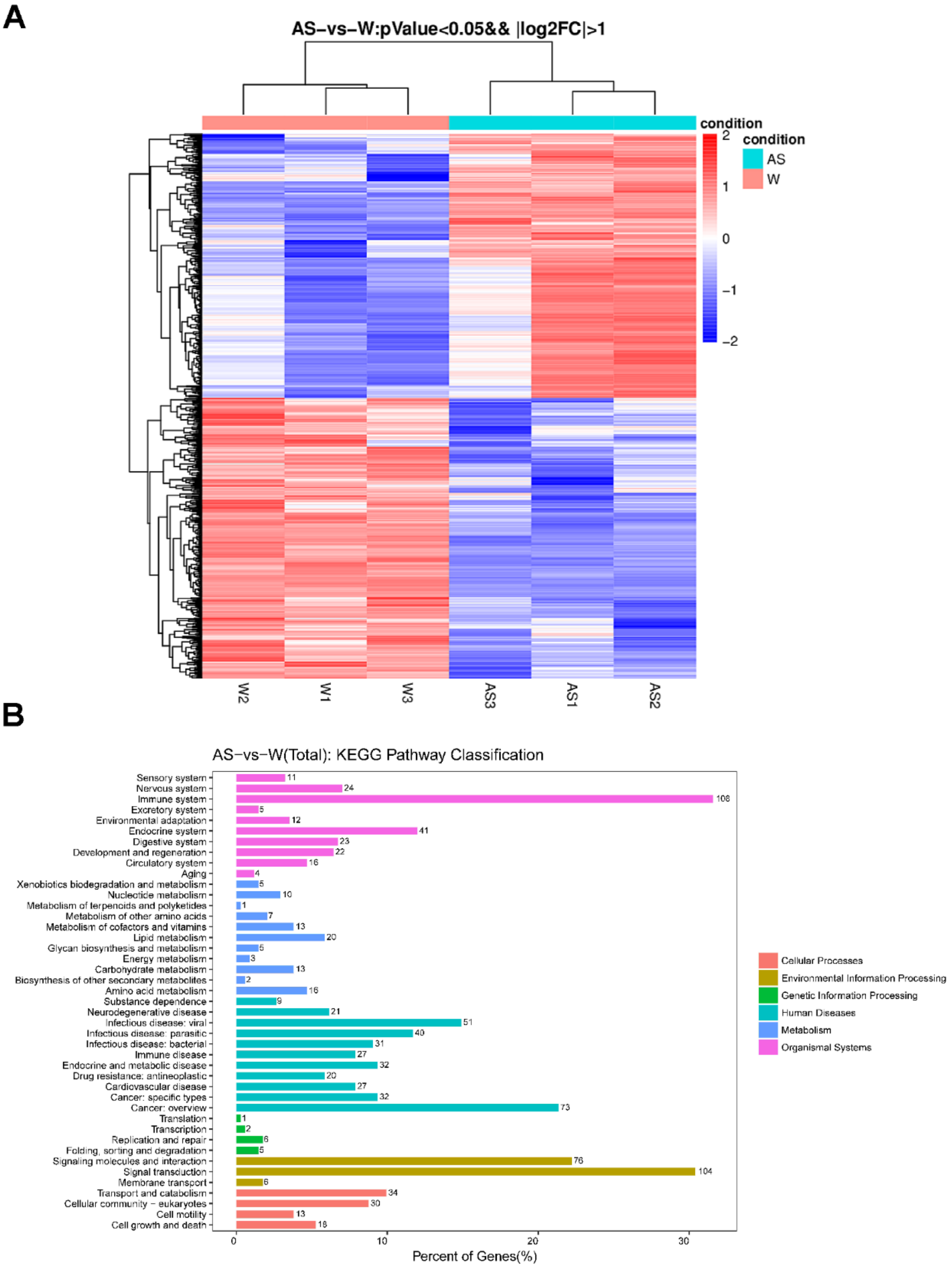

Supplementary Figure 5. (**A**). Heatmap of genes in CD4$^+$T cells with or without the astilbin treatment. (**B**). KEGG analysis.

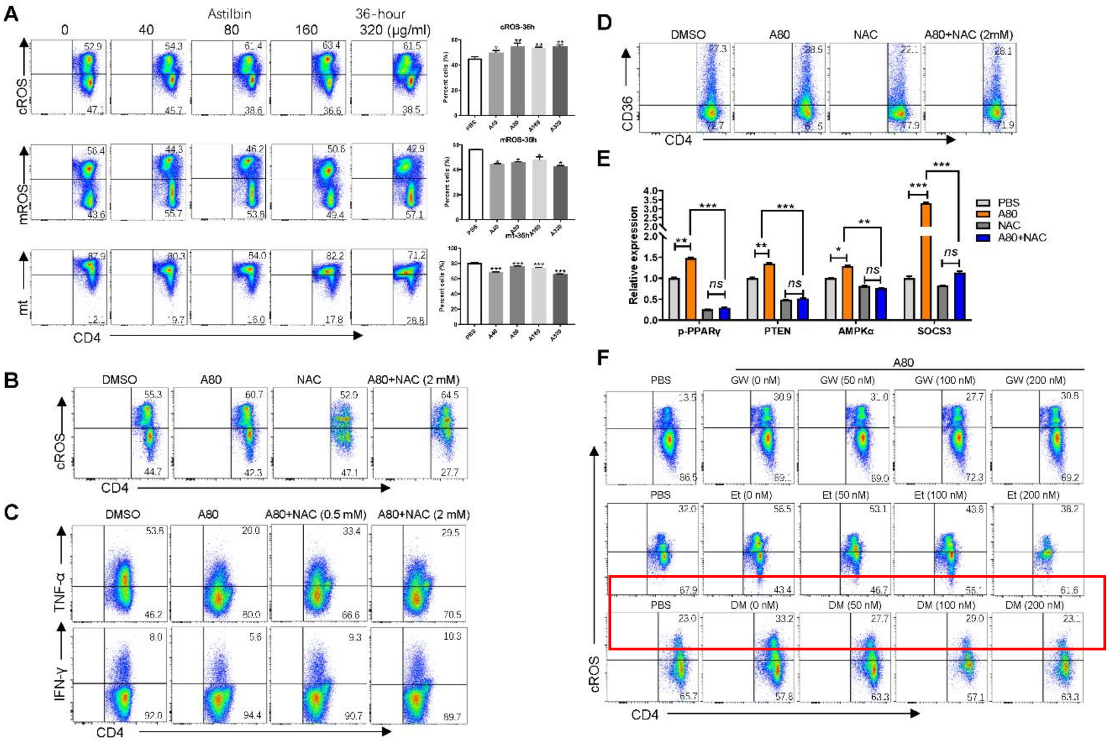

Supplementary Figure 6. (**A**). Production of cROS, mROS, and mitochondrial weight in CD4$^+$ T cells treated by astilbin for 36 hours. (**B**). Detection of cROS in astilbin-treated CD4$^+$ T cells by NAC. Representative results of TNF-α, IFN-γ (**C**) and CD36 (**D**) in astilbin-treated CD4$^+$ T cells with depletion of ROS. (**E**). ROS depletion on p-PPARγ, PTEN, AMPKα, and SOCS3 in astilbin-treated CD4$^+$ T cells. Mean±SD; n=3. (**F**). Production of cROS in astilbin-treated CD4$^+$ T cells after the CPT1α or AMPKα inhibition. All experiments were performed three times. P values (*$P \leq 0.05$; **$P \leq 0.01$; ***$P \leq 0.001$; ns, no significant difference) determined by one-way ANOVA (**E**).

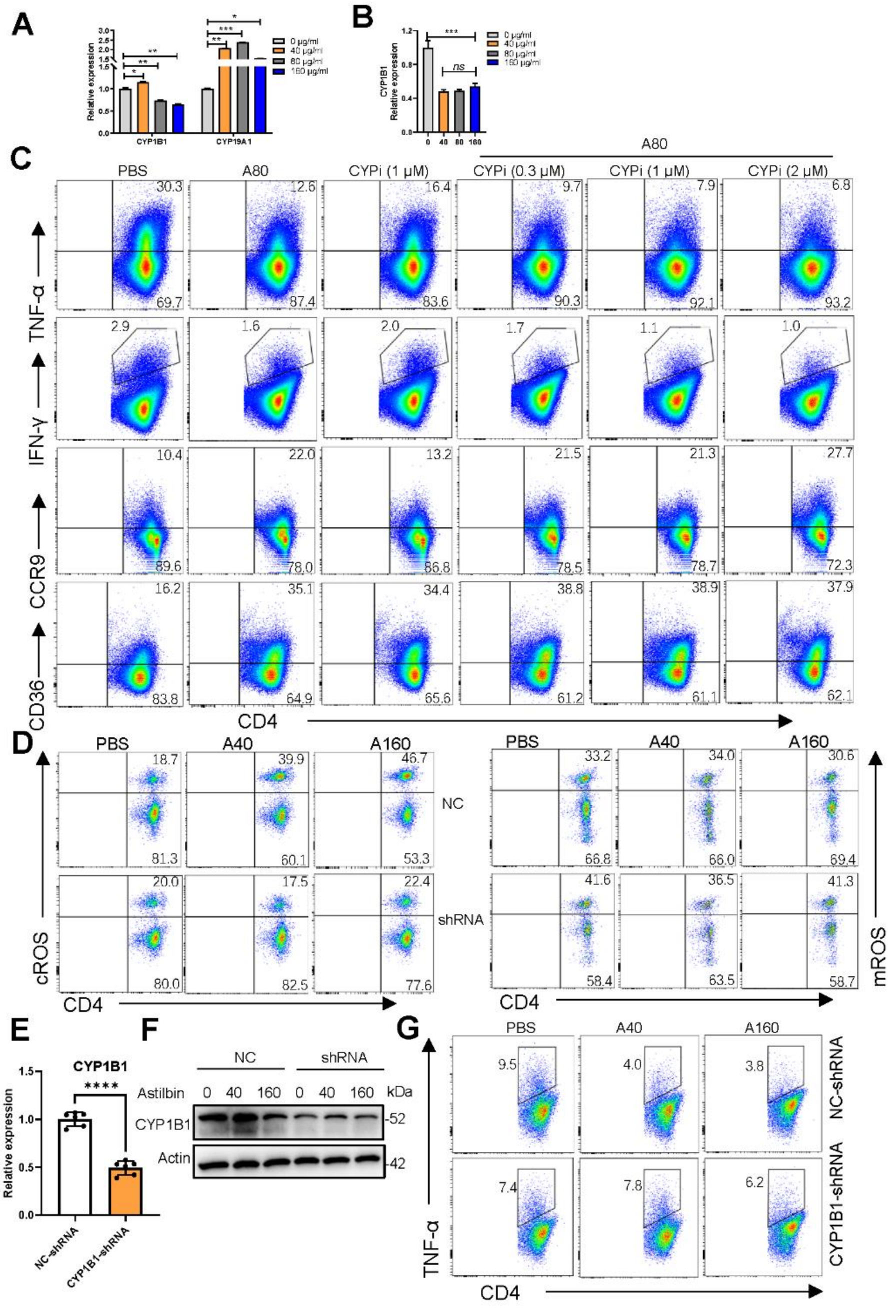

Supplementary Figure 7. (**A**). Statistics of CYP1B1 and CYP19A1 in astilbin-treated CD4$^+$ T cells by western blot. Mean±SD; n=3. (**B**). CYP1B1 mRNA level in astilbin-treated CD4$^+$T cells was analyzed by qRT-PCR. Mean±SD; n=3. (**C**). Representative results of TNF-α, IFN-γ, CD36 and CCR9 in astilbin-treated CD4$^+$ T cells after the CYP inhibition. (**D**). Production of cROS or mROS in CYP1B1 shRNA-transfected CD4$^+$ T cells by the astilbin treatment. CYP1B1 expression in CYP1B1 shRNA-transfected CD4$^+$ T cells detected by real-time PCR (**E**) and western blot (**F**). Mean±SD; n=3. (**G**). TNF-α production in CYP1B1 shRNA-transfected CD4$^+$ T cells by the astilbin treatment. All experiments were performed three times. P values (*$P \le 0.05$; **$P \le 0.01$; ***$P \le 0.001$; ns, no significant difference) determined by one-way ANOVA (**A**) and t-test (**B,E**)

Supplementary Table 1. DEGs associated with inflammation

Term	List Hits	List Total	Pop Hits	Pop Total	P Value	Q Value	Enrichment score	Gene
JAK-STAT signaling pathway	13	342	164	8203	0.00845	0.050475	1.90128	Csf2rb; Csf3r; Ifnlr1; Il10; Il12b; Il13ra1; Il2; Il21; Il6; Il7r; Il9r; Lif; Prlr
MAPK signaling pathway	20	342	294	8203	0.011078	0.058753	1.631659	Cacna1e; Cacna1g; Cacna2d2; Fgfr1; Fos; Hgf; Hspa1a; Hspa1b; Igf1; Il1b; Kit; Kitl; Map3k12; Mapk11; Mapk12; Mapk13; Pdgfc; Ptpn5; Rac3; Rasgrp4
TNF signaling pathway	9	342	108	8203	0.014375	0.071257	1.998782	Bcl3; Fos; Il1b; Il6; Lif; Mapk11; Mapk12; Mapk13; Mmp9

Supplementary Table 2. Candidate targets of astilbin

Target	Common name	Uniprot ID	Target Class	Probability*
Cyclooxygenase-1	PTGS1	P23219	Oxidoreductase	0.106542926
Cytochrome P450 19A1	CYP19A1	P11511	Cytochrome P450	0.106542926
P-glycoprotein 1	ABCB1	P08183	Primary active transporter	0.106542926
Carbonic anhydrase IV	CA4	P22748	Lyase	0.106542926
Carbonic anhydrase III	CA3	P07451	Lyase	0.106542926
Carbonic anhydrase VI	CA6	P23280	Lyase	0.106542926
Carbonic anhydrase VB	CA5B	Q9Y2D0	Lyase	0.106542926
Carbonic anhydrase VA	CA5A	P35218	Lyase	0.106542926
Carbonic anhydrase XII	CA12	O43570	Lyase	0.106542926
Tyrosyl-DNA phosphodiesterase 1	TDP1	Q9NUW8	Enzyme	0.106542926
Microtubule-associated protein tau	MAPT	P10636	Unclassified protein	0.106542926
Dual-specificity tyrosine-phosphorylation regulated kinase 1A	DYRK1A	Q13627	Kinase	0.106542926
HERG	KCNH2	Q12809	Voltage-gated ion channel	0.106542926
Beta amyloid A4 protein	APP	P05067	Membrane receptor	0.106542926
MAP kinase p38 alpha	MAPK14	Q16539	Kinase	0.106542926
Telomerase reverse transcriptase	TERT	O14746	Enzyme	0.106542926
6-phosphogluconate dehydrogenase	PGD	P52209	Enzyme	0.106542926
CMP-N-acetylneuraminate-beta-1,4-galactoside alpha-2,3-sialyltransferase	ST3GAL3	Q11203	Transferase	0.106542926
Alpha-(1,3)-fucosyltransferase 7	FUT7	Q11130	Transferase	0.106542926
Hepatocyte growth factor receptor	MET	P08581	Kinase	0.106542926
Matrix metalloproteinase 14	MMP14	P50281	Protease	0.106542926
Beta-secretase 1	BACE1	P56817	Protease	0.106542926
Apoptosis regulator Bcl-2	BCL2	P10415	Other ion channel	0.106542926
Fucosyltransferase 4	FUT4	P22083	Enzyme	0.106542926
Signal transducer and activator of transcription 1-alpha/beta	STAT1	P42224	Transcription factor	0.106542926
Squalene monooxygenase (by homology)	SQLE	Q14534	Enzyme	0.106542926

Carbonic anhydrase II	CA2	P00918	Lyase	0.106542926
Carbonic anhydrase I	CA1	P00915	Lyase	0.106542926
Steroid 5-alpha-reductase 1	SRD5A1	P18405	Oxidoreductase	0.106542926
Kallikrein 1	KLK1	P06870	Protease	0.106542926
Kallikrein 2	KLK2	P20151	Protease	0.106542926
Cytochrome P450 1B1	CYP1B1	Q16678	Cytochrome P450	0.106542926
Eukaryotic initiation factor 4A-I	EIF4A1	P60842	Hydrolase	0.106542926

Supplementary Table 3. Bonding types of CYP1B1 with astilbin under best conformation

Name	Distance	Category	Type
GLU229	2.29161	Hydrogen Bond	Conventional Hydrogen Bond
GLN340	2.24299	Hydrogen Bond	Conventional Hydrogen Bond
GLN340	2.86534	Hydrogen Bond	Pi-Donor Hydrogen Bond
PRO195	4.91698	Hydrophobic	Pi-Alkyl
VAL198	4.95401	Hydrophobic	Pi-Alkyl
ARG222	4.72842	Hydrophobic	Pi-Alkyl
ARG194	4.58186	Hydrophobic	Pi-Alkyl
VAL198	5.30853	Hydrophobic	Pi-Alkyl
LYS512	5.02613	Hydrophobic	Pi-Alkyl
