# Supplementary material for: Astilbin Activates the Reactive Oxidative Species/PPARγ Pathway to Suppress Effector CD4+ T Cell Activities via Direct Binding With Cytochrome P450 1B1
Source: Front Pharmacol. 2022 May 16;13:848957. doi: 10.3389/fphar.2022.848957 (PMC9150850; doi:10.3389/fphar.2022.848957)
Supplement: Supplementary file 8 [file DataSheet2.docx]

**Supplementary Table 1. DEGs associated with inflammation**

| Term | List  Hits | List  Total | Pop  Hits | Pop  Total | P  Value | Q  Value | Enrichment  score | Gene |
| --- | --- | --- | --- | --- | --- | --- | --- | --- |
| JAK-STAT signaling pathway | 13 | 342 | 164 | 8203 | 0.00845 | 0.050475 | 1.90128 | Csf2rb; Csf3r; Ifnlr1; Il10; Il12b; Il13ra1; Il2; Il21; Il6; Il7r; Il9r; Lif; Prlr |
| MAPK  signaling pathway | 20 | 342 | 294 | 8203 | 0.011078 | 0.058753 | 1.631659 | Cacna1e; Cacna1g; Cacna2d2; Fgfr1; Fos; Hgf; Hspa1a; Hspa1b; Igf1; Il1b; Kit; Kitl; Map3k12; Mapk11; Mapk12; Mapk13; Pdgfc; Ptpn5; Rac3; Rasgrp4 |
| TNF signaling pathway | 9 | 342 | 108 | 8203 | 0.014375 | 0.071257 | 1.998782 | Bcl3; Fos; Il1b; Il6; Lif; Mapk11; Mapk12; Mapk13; Mmp9 |

**Supplementary Table 2. Candidate targets of astilbin**

| Target | Common name | Uniprot ID | Target Class | Probability* |
| --- | --- | --- | --- | --- |
| Cyclooxygenase-1 | PTGS1 | P23219 | Oxidoreductase | 0.106542926 |
| Cytochrome P450 19A1 | CYP19A1 | P11511 | Cytochrome P450 | 0.106542926 |
| P-glycoprotein 1 | ABCB1 | P08183 | Primary active transporter | 0.106542926 |
| Carbonic anhydrase IV | CA4 | P22748 | Lyase | 0.106542926 |
| Carbonic anhydrase III | CA3 | P07451 | Lyase | 0.106542926 |
| Carbonic anhydrase VI | CA6 | P23280 | Lyase | 0.106542926 |
| Carbonic anhydrase VB | CA5B | Q9Y2D0 | Lyase | 0.106542926 |
| Carbonic anhydrase VA | CA5A | P35218 | Lyase | 0.106542926 |
| Carbonic anhydrase XII | CA12 | O43570 | Lyase | 0.106542926 |
| Tyrosyl-DNA phosphodiesterase 1 | TDP1 | Q9NUW8 | Enzyme | 0.106542926 |
| Microtubule-associated protein tau | MAPT | P10636 | Unclassified protein | 0.106542926 |
| Dual-specificity tyrosine-phosphorylation regulated kinase 1A | DYRK1A | Q13627 | Kinase | 0.106542926 |
| HERG | KCNH2 | Q12809 | Voltage-gated ion channel | 0.106542926 |
| Beta amyloid A4 protein | APP | P05067 | Membrane receptor | 0.106542926 |
| MAP kinase p38 alpha | MAPK14 | Q16539 | Kinase | 0.106542926 |
| Telomerase reverse transcriptase | TERT | O14746 | Enzyme | 0.106542926 |
| 6-phosphogluconate dehydrogenase | PGD | P52209 | Enzyme | 0.106542926 |
| CMP-N-acetylneuraminate-beta-1,4-galactoside alpha-2,3-sialyltransferase | ST3GAL3 | Q11203 | Transferase | 0.106542926 |
| Alpha-(1,3)-fucosyltransferase 7 | FUT7 | Q11130 | Transferase | 0.106542926 |
| Hepatocyte growth factor receptor | MET | P08581 | Kinase | 0.106542926 |
| Matrix metalloproteinase 14 | MMP14 | P50281 | Protease | 0.106542926 |
| Beta-secretase 1 | BACE1 | P56817 | Protease | 0.106542926 |
| Apoptosis regulator Bcl-2 | BCL2 | P10415 | Other ion channel | 0.106542926 |
| Fucosyltransferase 4 | FUT4 | P22083 | Enzyme | 0.106542926 |
| Signal transducer and activator of transcription 1-alpha/beta | STAT1 | P42224 | Transcription factor | 0.106542926 |
| Squalene monooxygenase (by homology) | SQLE | Q14534 | Enzyme | 0.106542926 |
| Carbonic anhydrase II | CA2 | P00918 | Lyase | 0.106542926 |
| Carbonic anhydrase I | CA1 | P00915 | Lyase | 0.106542926 |
| Steroid 5-alpha-reductase 1 | SRD5A1 | P18405 | Oxidoreductase | 0.106542926 |
| Kallikrein 1 | KLK1 | P06870 | Protease | 0.106542926 |
| Kallikrein 2 | KLK2 | P20151 | Protease | 0.106542926 |
| Cytochrome P450 1B1 | CYP1B1 | Q16678 | Cytochrome P450 | 0.106542926 |
| Eukaryotic initiation factor 4A-I | EIF4A1 | P60842 | Hydrolase | 0.106542926 |

**Supplementary Table 3. Bonding types of CYP1B1 with astilbin under best conformation**

| Name | Distance | Category | Type |
| --- | --- | --- | --- |
| GLU229 | 2.29161 | Hydrogen Bond | Conventional Hydrogen Bond |
| GLN340 | 2.24299 | Hydrogen Bond | Conventional Hydrogen Bond |
| GLN340 | 2.86534 | Hydrogen Bond | Pi-Donor Hydrogen Bond |
| PRO195 | 4.91698 | Hydrophobic | Pi-Alkyl |
| VAL198 | 4.95401 | Hydrophobic | Pi-Alkyl |
| ARG222 | 4.72842 | Hydrophobic | Pi-Alkyl |
| ARG194 | 4.58186 | Hydrophobic | Pi-Alkyl |
| VAL198 | 5.30853 | Hydrophobic | Pi-Alkyl |
| LYS512 | 5.02613 | Hydrophobic | Pi-Alkyl |
